# Dissecting the Molecular Origin of *g*-Tensor Heterogeneity and Strain in Nitroxide Radicals in Water: Electron Paramagnetic Resonance Experiment versus Theory

**DOI:** 10.1021/acs.jpca.3c02879

**Published:** 2023-07-31

**Authors:** Van Anh Tran, Markus Teucher, Laura Galazzo, Bikramjit Sharma, Tim Pongratz, Stefan M. Kast, Dominik Marx, Enrica Bordignon, Alexander Schnegg, Frank Neese

**Affiliations:** † Max-Planck-Institut für Kohlenforschung, Kaiser-Wilhelm-Platz 1, 45470 Mülheim an der Ruhr, Germany; ‡ Max-Planck-Institut für Chemische Energiekonversion, Stiftstraße 34-36, 45470 Mülheim an der Ruhr, Germany; § Department of Physical Chemistry, University of Geneva, Quai Ernest Ansermet 30, 1211 Geneva, Switzerland; ∥ Faculty of Chemistry and Biochemistry, Ruhr-Universität Bochum, 44780 Bochum, Germany; ⊥ Lehrstuhl für Theoretische Chemie, Ruhr-Universität Bochum, 44780 Bochum, Germany; @ Fakultät für Chemie und Chemische Biologie, Technische Universität Dortmund, Otto-Hahn-Str. 4a, 44227 Dortmund, Germany

## Abstract

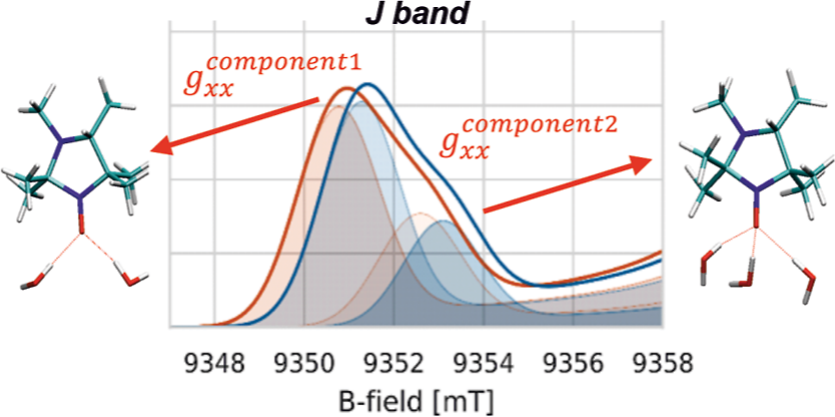

Nitroxides are common EPR sensors of microenvironmental properties such as polarity, numbers of H-bonds, pH, and so forth. Their solvation in an aqueous environment is facilitated by their high propensity to form H-bonds with the surrounding water molecules. Their *g*- and *A*-tensor elements are key parameters to extracting the properties of their microenvironment. In particular, the *g*_*xx*_ value of nitroxides is rich in information. It is known to be characterized by discrete values representing nitroxide populations previously assigned to have different H-bonds with the surrounding waters. Additionally, there is a large *g*-strain, that is, a broadening of *g*-values associated with it, which is generally correlated with environmental and structural micro-heterogeneities. The *g*-strain is responsible for the frequency dependence of the apparent line width of the EPR spectra, which becomes evident at high field/frequency. Here, we address the molecular origin of the *g*_*xx*_ heterogeneity and of the *g*-strain of a nitroxide moiety (HMI: 2,2,3,4,5,5-hexamethylimidazolidin-1-oxyl, C_9_H_19_N_2O_) in water. To treat the solvation effect on the *g*-strain, we combined a multi-frequency experimental approach with ab initio molecular dynamics simulations for structural sampling and quantum chemical EPR property calculations at the highest realistically affordable level, including an explicitly micro-solvated HMI ensemble and the embedded cluster reference interaction site model. We could clearly identify the distinct populations of the H-bonded nitroxides responsible for the *g*_*xx*_ heterogeneity experimentally observed, and we dissected the role of the solvation shell, H-bond formation, and structural deformation of the nitroxide in the creation of the *g*-strain associated with each nitroxide subensemble. Two contributions to the *g*-strain were identified in this study. The first contribution depends on the number of hydrogen bonds formed between the nitroxide and the solvent because this has a large and well-understood effect on the *g*_*xx*_-shift. This contribution can only be resolved at high resonance frequencies, where it leads to distinct peaks in the *g*_*xx*_ region. The second contribution arises from configurational fluctuations of the nitroxide that necessarily lead to *g*-shift heterogeneity. These contributions cannot be resolved experimentally as distinct resonances but add to the line broadening. They can be quantitatively analyzed by studying the apparent line width as a function of microwave frequency. Interestingly, both theory and experiment confirm that this contribution is *independent* of the number of H-bonds. Perhaps even more surprisingly, the theoretical analysis suggests that the configurational fluctuation broadening is *not* induced by the solvent but is inherently present even in the gas phase. Moreover, the calculations predict that this broadening *decreases* upon solvation of the nitroxide.

## 1 Introduction

Nitroxide radicals are important probe molecules in organic chemistry and biochemistry.^1^ The unpaired electron (*S* = 1/2) localized in the N–O moiety is usually stabilized by the presence of bulky alkyl groups in the α-position to the nitrogen atom. Specific nitroxides are employed as radical traps, which can be added to liquid solutions to trap and identify more short-lived radicals, for example, reactive oxygen species or carbon-centered radicals.^2^ In addition, nitroxides are widely used as site-specific spin labels in structural biology to measure distances between protein subunits^3,4^ or to probe local properties like water accessibility, flexibility of sites,^5−7^ and the formation of hydrogen bonds (H-bonds).^8−11^ Nitroxides can also be used as in situ reporters of pH or redox properties because modification of the basic structure of the nitroxide can tune its specific response to different microenvironments, as summarized here.^12^

Local properties of nitroxides can be studied in detail via line shape analyses of their EPR spectra.^8^ Particularly, relevant for the study of environmental influences are the characteristic nitroxide ^14^N hyperfine tensor (^14^N *A*-tensor, main values *A*_*xx*_, *A*_*yy*_, and *A*_*zz*_) and the anisotropic Zeeman interaction, parameterized by the *g*-tensor (main values *g*_*xx*_, *g*_*yy*_, and *g*_*zz*_). The *g*_*xx*_ and *A*_*zz*_ components are sensitive to changes in the local polarity of the medium surrounding the nitroxide probe.^11^ While *g*_*xx*_ values decrease in polar/protic environments, *A*_*zz*_ (and *A*_iso_) increase.^9,10^ This property has been used to investigate protein channels and probe differences in the nitroxide microenvironment in different solvents.^13^ Beyond the polarity information, the propensity to form H-bonds can be detected via the *g*_*xx*_ parameter. For nitroxides in frozen aqueous solutions, distinct spectral fractions characterized by different *g*_*xx*_ parameters were observed, which were attributed to different degrees of H-bonding (see for example^11,14,15^). The thermal history of the sample was also found to be important in determining the ratio of the different spectral components. In fact, variations in the relative fractions of the *g*_*xx*_ component were observed upon annealing at the glass transition temperature of water-glycerol mixtures.^13^

Nitroxide *g*- and *A*-values can be accurately determined by calculation of the nitroxide EPR spectrum by a spin-Hamiltonian (SH) approach and fitting of the calculated spectrum to the experiment. For freely tumbling nitroxides in a liquid solution, *g*- and *A*-tensor anisotropies are averaged. In this case, the EPR line shape is determined by the isotropic *A*- and *g*-values *A*_iso_ and *g*_iso_, respectively. On the contrary, in frozen solutions, one obtains a powder pattern where, due to the orientation dependence of the *A*- and *g*-tensors, different orientations of the nitroxide with respect to the external magnetic field appear at characteristic field positions. The resulting spectra strongly depend on the chosen resonance condition. In the case of nitroxides, conventional X-band EPR (9.5 GHz/0.34 T) spectra are mainly influenced by the *A*-tensor anisotropy, in particular the dominating *A*_*zz*_ value. To exploit the information contained in the *g*-tensor, higher fields/frequencies are required to resolve its anisotropy (typically ≥ 95 GHz/3.4 T, see Supporting Information Section 1 and Figure S1).

**Figure 1:**
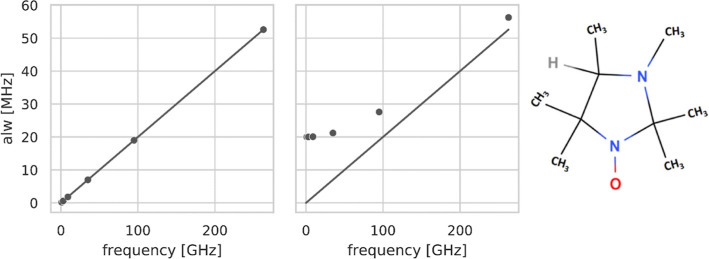
Schematic plot of apparent line width (*alw*) vs resonance frequency for organic radicals adapted from Hagen.^23^ In both panels, the intrinsic *lw* is considered negligible (y-intercept equal to zero). Left, the frequency dependence of the *alw*, for example, organic radical in the absence of field-independent line broadening. Right, frequency dependence of the *alw* for a more common case in which a field-independent line width of 20 MHz (0.7 mT) is predominant below 95 GHz/3.5 T, and the subsequent linear increase is due to frequency-dependent *g*-strain effects. The straight line represents the field-dependent component of the *alw*. On the right is the radical investigated in this study (neutral form of HMI). Its protonated form (HMIH^+^) is not investigated here.

In terms of the apparent line width (*alw*) observed for each orientation of the molecule with respect to the magnetic field, one can distinguish at least three components: (1) the intrinsic line width. This is the natural line width of the EPR resonance, which is, of course, intimately tied to electron spin relaxation times. Nitroxides in frozen water-glycerol solutions at around 100 K exhibit relaxation times in the single-digit μs range,^16^ which corresponds to intrinsic line widths below 1 MHz. (2) The *field-independent broadening* resulting from small, unresolved hyperfine and quadrupole interactions of weakly coupled nuclei in the surrounding. These can come from the solute itself or also from the magnetic nuclei of the solvent, for example, solvent protons. Importantly, as one raises the resonance field/frequency, this broadening mechanism does not lead to a further increase of the *alw*. (3) The *field-dependent broadening*. It is generally agreed that this broadening mechanism is related to micro-heterogeneity in the molecular ensemble in frozen solutions. The heterogeneous environment leads to slightly different *g*-values (and zero-field splitting in high-spin systems) for different members of the ensemble, which consequently resonate at different frequencies. Since at higher microwave frequencies, these resonances will split apart, this mechanism leads to an increase in the *alw* with frequency. The latter phenomenon is referred to as the *g*-strain.^17^ This increase is believed to be linear in the microwave frequency (Figure 1).^17−19^

Of course, there is the possibility that by increasing the microwave frequency further, one starts to resolve subensembles of the solute in discrete resonances, with each subensemble characterized by its own *g*-strain. It has been shown that the *g*-strain depends on the structure of the host molecule and the solvent properties, as the *g*-strain of radical cofactors in well-defined protein binding pockets was found to be much less pronounced than for the same radicals in frozen solutions.^20,21^ For the case of nitroxides, field-dependent line broadening was assigned to *g*-tensor variations via site-to-site variations of the local environment^15^ and nitroxide structure.^22^ This *g*-strain was found to be most pronounced for the nitroxide *g*_*xx*_ values.^10,11,15,22^ Experimentally observed variations of *g*_*xx*_ on the order of 5*10^–4^ were assigned to a varying number of hydrogen bonds, leading to a heterogeneous *g*_*xx*_ region,^10,11,15^ while the polarity of the solvent had little effect on the *g*-strain^15^ (see Table 1 for a small summary of *g*- and *A*-values based on studies of nitroxides in different solvents).

**Table 1:** Summary of Some *g*- and *A*-Tensor Parameters Available in the Literature Obtained with Nitroxide Probes in Different Solvents^a^

H-bonds	nitroxide/solvent	(*g*_*xx*_, *g*_*yy*_, *g*_*zz*_) – 2/10^–^^5^	*A*_*xx*_, *A*_*yy*_, *A*_*zz*_ [MHz]	(*g*_*xx*_*–g*_*zz*_)/10^–^^5^	refs
0	pyrroline-type nitroxide (R1)/2-propanol (***Comp1***)	**910.9**; 619.6; 218.5	12.07; 12.07; **93.16**	∼690	(22)
1	R1/2-propanol (***Comp2***)	**843.2**; 606.2; 218.5	13.24; 13.30; **99.68**	∼620	(22)
0	R1/ortho-terphenyl	**905.0**; 615.0; 219.8	12.10; 12.18; **94.67**	∼690	(22)
1	R1–OH calculated	**845.3**, 605.0, 215.5		∼630	(22)
0	R1 calculated	**890.0**, 624.8, 211.8		∼670	(22)
0	R1/OTP	**910.0**	**94.7**	∼700*	(15)
1	R1/aniline	**877.0**	**98.3**	∼670*	(15)
1	R1/phenol (***Comp1***)	**820.0**	**103**	∼610*	(15)
2	R1/phenol (***Comp2***)	**768.0**	**109**	∼560*	(15)
0	R1/2-propanol (***Comp1***)	**923.0**	**93.8**	∼710*	(15)
1	R1/2-propanol (***Comp2***)	**855.0**	**99.4**	∼640*	(15)
1	R1/methanol	**844.0**	**101**	∼630*	(15)
0	R1/glycerol (***Comp1***)	**907.0**			(15)
1	R1/glycerol (***Comp2***)	**846.0**	**101**	∼640*	(15)
2	R1/glycerol (***Comp3***)	**800.0**		∼590*	(15)
1	R1/water (***Comp1***)	**842.0**	**101**	∼630*	(15)
2	R1/water (***Comp2***)	**800.0**	**106**	∼590*	(15)

a In bold, we highlighted the *g*_*xx*_ parameters attributed to spectral components (denoted as “***Comp#***” in the following) derived from distinct configurations having 0, 1, or 2 H-bonds and the corresponding *A*_*zz*_ components.

However, a deep-reaching analysis of the molecular origin of *g*-strain is missing so far. It should be noted that due to a lack of information on the local properties of the probe molecules, modeling of field-dependent line broadenings often assumes simple Gaussian distributions of the main *g*-values.

The contributions to the *alw* as a function of frequency for an organic radical are shown in Figure 1 (adapted from Hagen^23^). The left plot shows the frequency dependence of the *alw* due to pure *g*-strain. The right plot shows the dependence of the *alw* when both field-independent and field-dependent contributions are present. Considering the intrinsic line width to be negligible, the overall *alw* is calculated as
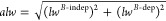
1

At low frequencies, the field-independent broadening contributions dominate, thus leading to an essentially constant value of the *alw* as a function of frequency. However, at a defined microwave frequency, the field-dependent broadening mechanisms start to dominate, and the subsequent increase of the *alw* with frequency is linear.^17−19^ The *alw* vs. *frequency* plot allows determining both the field-dependent and the field-independent contributions (non-resolved hyperfine interactions and intrinsic line width). Since the intrinsic line width can be determined through relaxation measurement, the remaining field-independent contributions to the *alw* might be extracted.

In this paper, we are mostly interested in understanding the molecular origin of the contributions to the field-dependent broadening mechanisms by using a combination of multi-frequency EPR spectroscopy, ab initio molecular dynamics (AIMD) simulations, and EPR property calculations at the highest realistically affordable quantum chemical level.

The field of theoretical spectroscopy has been very active in recent years (for reviews, see refs (24)–^32^). The challenges under investigation are manifold and include the explicit treatment of solvent effects for systems of nontrivial size,^31,33−39^ the development and validation of improved density functionals^40−46^ and correlated ab initio methods,^34,37,47−51^ the treatment of relativistic-^51,52^ and vibronic-^50,53,54^ effects as well as conceptual and algorithmic advances^36,38,43−46,48,51,55−58^ to name only a few. The characterizing parameters of a nitroxide’s EPR spectrum, namely, the *g*- and *A*-tensors, are accessible at different levels of theory.^29^ While many studies are based on density functional theory (DFT),^59−65^ the development of electron correlation methods gives access to more accurate predictions. Recent advances in local correlation theory, such as the DLPNO-CCSD method,^66−69^ enable coupled cluster-level results of *A*-tensors for larger and more complex systems, for instance, molecular clusters of an organic radical surrounded by a fair number of solvent molecules, as we previously did for an HMI molecule in water.^70^ However, the *g*-tensor being a second-order property is, unfortunately, not yet available at this level of theory. Nonetheless, DFT-based calculations, in particular hybrid DFT, have shown good performance for small- to medium-sized organic molecules.^31,71^ Different theoretical studies have been performed to investigate the effect of polarity and H-bonding on the magnetic tensors,^9,10,72,73^ but there are no theoretical studies addressing the molecular origin of the different spectral components in aqueous solutions or the origin of the *g*-strain, which is experimentally observed.

Previously, we studied the pH-sensitive HMI spin probe (2,2,3,4,5,5-hexamethylimidazolidin-1-oxyl, C_9_H_19_N_2_O) in its neutral unprotonated form (Figure 1) and used an ensemble of state-of-the-art computational techniques to obtain the isotropic hyperfine coupling constant to be compared with that extracted from X-band continuous wave (cw) EPR spectra detected at room temperature in aqueous solution.^70^ Our aim was to delineate the cutting edge of current first-principles-based calculations of EPR parameters in aqueous solutions based on rigorous statistical mechanics combined with correlated electronic structure techniques.^70^ This probe is attractive for such a combined theoretical–experimental study because it offers the possibility to explore the solvation-induced changes in the *g*- and *A*-values in two different ground states, namely, in the unprotonated and protonated forms, which widens the scope of the theoretical–experimental comparison.

Here, we extend the previous work by investigating the molecular determinants of the *g*_*xx*_ heterogeneity and the associated *g*-strain of the neutral unprotonated HMI by comparing theoretical predictions with multi-frequency experimental data obtained at cryogenic temperature. This analysis provides the basis for understanding the solvation-induced and HMI-induced g variations and paves the way for further exploration of the more challenging protonated HMIH^+^ nitroxide radical in aqueous solutions in future work.

## 2 Experimental Details

### 2.1 Sample Preparation

The HMI spin probe (2,2,3,4,5,5-hexamethylimidazolidin-1-oxyl, C_9_H_19_N_2_O) was synthesized as described in.^70,74−76^ For the EPR experiments, solutions containing 500 micromolar HMI were prepared in ultra-pure milli-Q water. NaOH at a final concentration of 0.1 mM was added (pH = 10 measured with a FiveEasy Plus FP20 pH-meter from Mettler Toledo) to ensure that the unprotonated HMI form was predominant, as shown before^70^ (the p*K*_A_ of HMI is about 4.5 at ambient temperature^77^). 10% (v/v) d-glycerol was added as a cryoprotectant to the samples, which were inserted in the EPR tubes and shock-frozen in liquid nitrogen.

### 2.2 EPR Experimental Parameters

cw X-band EPR (9.8 GHz/0.35 T) experiments were performed using a Bruker ELEXSYS E580 (Rheinstetten, Germany) spectrometer in conjunction with a Bruker MD-5 resonator. The spectra were acquired at 100 K with 0.013 mW microwave power, a modulation amplitude of 0.1 mT, a conversion time of 40 ms, a field sweep of 20 mT, and 1024 points.

Q-band EPR (33.7 GHz/1.2 T) experiments were performed using the same spectrometer with a custom-built Q-band accessory and a Bruker CW resonator. The spectra were acquired at 100 K, with 4 mW microwave power, a modulation amplitude of 0.5 mT, a conversion time of 40 ms, a field sweep of 20 mT, and 1024 points. 32 spectra were averaged to optimize the signal-to-noise ratio.

For the W-band EPR (94 GHz/3.35 T) measurements, a modified Bruker ELEXSYS II E680 spectrometer. The spectra were acquired at 100 K with 5.6*10^–5^ mW microwave power, a modulation amplitude of 0.3 mT, a conversion time of 81.92 ms, a field sweep of 60 mT, and 8192 points.

The J-band EPR (262.8 GHz/9.38 T) data were acquired with a Bruker ELEXSYS 780 spectrometer equipped with a non-resonant sample insert. The spectra were acquired at 100 K, with 1.5 mW microwave power, a modulation amplitude of 1 mT, a conversion time of 800 ms, a field sweep of 50 mT, and 2024 points. The modulation amplitude for lock-in detection of the CW EPR spectra was adjusted for each frequency band to achieve optimum sensitivity and, at the same time, guarantee optimum resolution of the spectral features.

### 2.3 EPR Data Analysis

The multi-frequency EPR spectra were post-processed using a frequency- and spectrometer-specific magnetic field calibration, as described in Supporting Information Section 3. Subsequent spectral simulations were carried out with the MATLAB routine “pepper” from EasySpin (version 5.2.33),^78^ employing the following SH
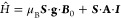
2where the first term denotes the electron Zeeman interaction, which couples the electron spin ***S*** to the external magnetic field ***B***_0_. The second term is the hyperfine interaction, which couples ***S*** and ***I*** the ^14^N nuclear spin of the N–O moiety (*I* = 1). **g** and **A** are the *g*-tensor and hyperfine tensor, respectively. (Note that we use the commonly accepted term “*g*-tensor” despite the fact that the *g*-matrix is not symmetric;^29,79^ the distinction is of no consequence for the findings discussed in this work). Since the influence of the nuclear Larmor frequency and the ^14^N quadrupole interaction could not be resolved in the multi-frequency EPR spectra, they were neglected in the simulations. EPR powder spectra are calculated in EasySpin employing matrix diagonalization of the SH in eq 2 over a triangular orientational grid. An orientation-dependent phenomenologically inhomogeneous Gaussian line broadening was considered (referred to as *alw*, with distinct line widths *alw*_*xx*,_*alw*_*yy*_, and *alw*_*zz*_ for the *x*, *y,* and *z* direction of the molecular frame, respectively), as specified in EasySpin with the function HStrain. Both field-dependent and field-independent Gaussian line broadenings were modeled by one set of orientation-dependent line widths. All broadenings are given as full width and half-maximum values. ^14^N *A*- and *g*-tensors were obtained by simultaneous fits to the X-, Q-, W-, and J-band EPR spectra. As will be explained below, two different *g*_*xx*_ populations were necessary to satisfactorily fit the experimental spectra. In these fits, only the *phenomenological* orientation-dependent inhomogeneous line widths were allowed to vary for different frequencies. In this way, we aim to not prejudice the analysis in any way by making assumptions about the broadening mechanism, which may or may not hold. The obtained “simulated spectrum” of the experiment (**exp**) resulted in the final spectrum that we will refer to as “**exp-sim**”, whereas the two underlying spectra distinguished by different *g*_*xx*_ values are named “***Comp1***” and “***Comp2***”. The **exp-sim** spectrum is the result of the data reduction process leading from the primary observation to a set of SH and line width parameters. The small deviations from the experiment **exp** arise from the sum of all the small interactions in the SH that were not modeled. Importantly, as will be detailed below, the theoretical spectra obtained by summing the simulated traces from each snapshot or subensemble thereof have been treated in the same way, thus allowing for an unbiased comparison of the theory and experiment.

## 3 Theoretical/Computational Details

### 3.1 Ab Initio Molecular Dynamics

An AIMD^80^ protocol, as developed, validated, and standardized previously,^70^ was used to sample the solvation configurational space of HMI in bulk water at ambient conditions. We refer the interested reader to that source for comprehensive background and details, but summarize here the key aspects: we performed AIMD simulations of one HMI molecule in 128 water molecules, all hosted in a periodically replicated cubic simulation box of dimension 15.9581 Å thermostated at 300 K. To cope with open-shell EPR spin probe molecules, we use a spin-polarized hybrid functional, namely, revPBE0^81,82^ in conjunction with the D3 dispersion correction,^83^ using its two-body terms with zero damping. The AIMD simulations were performed using the CP2K software package,^84,85^ and the auxiliary density matrix method^86^ was employed to speed up the computation of the Fock matrix in the hybrid functional. The initial configuration to launch AIMD sampling was generated by force field molecular dynamics simulations using the Gromacs simulation package,^87^ the corresponding details of which are reported in the Supporting Information of ref (70).

For AIMD simulation of HMI in vacuum, we have adopted the same protocol described for solvated HMI except that non-periodic cluster boundary conditions were employed while again thermostating at 300 K. Hence, the wavelet Poisson solver^88,89^ was used, which is appropriate for non-periodic boundary conditions.

From a total AIMD trajectory of 206 ps lengths both in the liquid state and vacuum, the first 6 ps equilibration part was discarded, and the subsequent 200 ps part was used to extract solvated and gas phase snapshots of HMI to conduct the single-point quantum chemical computations of EPR properties for these configurations. From these trajectories, two ensembles of 1000 snapshots for the liquid and gas phases were obtained to carry out quantum chemical calculations with revPBE0-D3 functional, whereas only 400 snapshots were extracted after every 500 fs in the liquid state to carry out computationally demanding DLPNO-CCSD calculations.

Hence, we generated the following ensembles from two distinct AIMD trajectories which are subjected to property calculations:

- Solvated AIMD: “solv-set1000”, containing 1000 snapshots of HMI in water, “solv-set400”, containing 400 snapshots of HMI in water.
- Vacuum AIMD: “vac-set1000”, containing 1000 snapshots of the HMI in vacuum.

### 3.2 Quantum Chemical Calculations at AIMD Snapshots

Quantum chemical single-point property calculations were performed for each snapshot which was extracted from the AIMD trajectories using a development version of the ORCA program package based on ORCA version 5.0.^90^ The *g*- and *A*-values were calculated at revPBE0ech Eds about (81)/def2-TZVPP-deconS level either on the fully solvated snapshot (solv-set1000-QM/MM) or on the vertically desolvated HMI (“solv-set1000-vd”), that is, removing all solvent molecules prior to the calculation. The hybrid quantum-mechanical/molecular-mechanical (QM/MM) scheme, as described in our previous publication,^70^ was applied to the fully solvated structures; that is, the QM region was calculated at the described level of theory, and the MM region was included as TIP3P^91^ point charges. The *A*-values for solv-set400-QM/MM were computed at the DLPNO-CCSD^66,69^/def2-TZVPP-deconS level in the QM regime. For the *g*-tensor calculations, the center of spin density^58^ was chosen for the gauge origin treatment. The vac-set1000 was treated at the same level of theory as the solv-set1000-QM/MM and solv-set1000-vd.

### 3.3 Embedded Cluster Reference Interaction Site Model

Embedded cluster reference interaction site model (EC-RISM) calculations were performed analogously to the calculations described in.^70^ Analogous to the QC calculations, the revPBE0/def2-TZVPP-deconS and DLPNO-CCSD/def2-TZVPP-deconS levels of theory, respectively, were used in conjunction with the PSE-3 closure, denoted solv-set1000-EC-RISM and solv-set400-EC-RISM. The solvent susceptibility from ref (93) was used. All other settings can be found in ref (70). Before the EC-RISM calculations were performed, all explicit waters were removed from the snapshots; that is, the structures were vertically desolvated.

### 3.4 H-bond Analysis along the AIMD Trajectory

The solvated trajectory was separated into H-bond subensembles using the same H-bonding criterion as parameterized and used previously for pure bulk water,^94^ namely, *r*_O···H_ < −1.71 cos θ + 1.37, but now involving the oxygen site of HMI (O) and of the water molecules as the acceptor and donor sites, respectively. This is justified since the same parameterization as for water–water H-bonding is found to properly describe HMI–water. This criterion will thus be used to analyze the structural properties of HMI solvation in aqueous solution as well as to disentangle the EPR spectra with respect to different H-bonding patterns of HMI using their populations. A detailed explanation is given in Supporting Information Section 4 in conjunction with the geometrical parameters shown in Figure 5. Such hydrogen bond analysis was performed on the HMI in water AIMD trajectory (see Section 4.2.1) to obtain one, two, and three hydrogen-bonded subensembles, which will be used to analyze theoretical spectra, as outlined in the next Section 3.5.

### 3.5 Theoretical EPR Data Analysis

The theoretical multicomponent approach to simulating the EPR spectra is based on our H-bond analysis along the solvated HMI ensembles solv-set1000 or solv-set400. For each of the subensembles, the mean *g*- and *A*-values, that is, averaged over the snapshots of the corresponding H-bond subensemble, were determined to simulate a spectrum for each subensemble. Those spectra are referred to as “***TComp#***”, with the number # referring to the corresponding number of H-bonds in the respective subensemble. The simulated spectrum is the weighted sum of the ***TComp#*** spectra. The weights used in the simulations are derived from the population analysis of the different H-bond subensembles along the AIMD trajectory.

### 3.6 Workflow of Theoretical *g*-Strain Analysis

**Figure 2:**
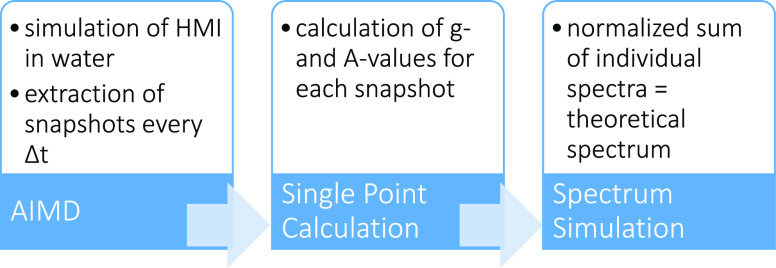
Visualization of the workflow that results in what will be referred to as the “theoretical spectrum” in the text with Δ*t* = 200 fs for solv-set1000 and Δ*t* = 500 fs for solv-set400.

The workflow to generate the EPR spectrum from the computed *g*- and *A*-values is depicted in Figure 2: for each snapshot that is extracted from the AIMD trajectory, a property calculation is performed either using the established QM/MM scheme or applying EC-RISM for the treatment of solvation. This resulted in a set of *g*- and *A*-values for each snapshot, for which a spectrum was simulated using the “pepper” routine in EasySpin.^78^

Assuming that the orientation and frequency dependence of the inhomogeneous line widths solely originate from variations in the *g*-tensor, a single field and orientation-independent inhomogeneous Gaussian line width of 17 MHz was employed in the simulations of the theoretical spectra. This value was estimated from the *alw* of the experimental X-band EPR spectrum (see Table 2) and accounts for the field-independent component of the *alw* in the simulation of the theoretical spectra, which cannot be otherwise extracted from our calculations. Multi-frequency EPR spectra were calculated with the same microwave frequencies as used in the experimental measurements (X-band: 9.7671 GHz, Q-band: 33.6615 GHz, W-band: 93.993 GHz, and J-band: 262.8436 GHz). The normalized sum of all single spectra resulted in what is referred to as the “theoretical spectrum”.

**Table 2:** Parameters Used for the Fitting of the Experimental (Multi-frequency) Spectra^a^

		**exp-sim**^b^	
		***Comp1***	***Comp2***
weights		0.67	0.33
*g*_*xx*_		2.00834	2.00795
(*g*_*xx*_–*g*_*zz*_)/10^–^^5^		604	565
*g*_*yy*_		2.00598	
*g*_*zz*_		2.0023	
*A*_*xx*_ [MHz]		14	
*A*_*yy*_ [MHz]		14	
*A*_*zz*_ [MHz]		100	
*alw*_*xx*_ [MHz]	X	20	
	Q	24	
	W	24	
	J	52	
*alw*_*yy*_ [MHz]	X	20	
	Q	22	
	W	21	
	J	28	
*alw*_*zz*_ [MHz]	X	18	
	Q	18	
	W	18	
	J	25	

a Two *g*_*xx*_ components were used in the fit, denoted with the columns ***Comp1*** and ***Comp2***. An orientation-dependent phenomenological Gaussian line broadening was considered (distinct line widths *alw*_*xx*,_*alw*_*yy*_, and *alw*_*zz*_), as specified in EasySpin with the function HStrain.

b All parameters are obtained by simulation with EasySpin of the experimental spectra using a global fit to the multi-frequency EPR data. The quality of the fits is evaluated by visual inspection of the match between experiment and simulation. The main sources of error for this procedure are the limited signal-to-noise ratio of the experimental data and inaccuracies in the calibration of the magnetic field. In the present study, a major effort was made to calibrate the magnetic field by comparing the results with two very accurate EPR field standards (see the Supporting Information). From these procedures, the absolute errors of the values reported in Table 2 were estimated to be ∼±1 10^–5^ for the *g*-values and ∼±1 MHz for the hyperfine value.

For comparison with the experimentally obtained SH parameters and the *g*-strain analysis, the so-obtained spectra were further treated completely analogously to the experimental spectra as described in Section 2.3. This amounts to performing simulations and least square fits in EasySpin based on a multicomponent Ansatz to fit *g*- and *A*-tensors as well as line widths to either the experimental or theoretical spectrum. In addition to obtaining apparent *g*- and *A*-values, the most important outcome of this fit is the apparent orientation-dependent line width as a function of microwave frequency. According to eq 1, the field-dependent line width component was extracted from the *alw*, which was then subjected to a linear regression fit. The slope of this fit serves as a quantification of the *g*-strain. This data then forms the basis for the comparison of experimental and theoretical *g*-strain parameters.

## 4 Results and Discussion

The results section is separated into different parts. First, we provide the experimental data and its analysis since the experiment serves as the reference point for the theoretical investigation. Afterward, we focus on the analysis of the computed EPR parameters, in particular the *g*-values and the hyperfine coupling constants of the nitroxy ^14^N-nucleus (for simplicity referred to as “*A*-values” in the following). Finally, we investigate the *g*-strain phenomenon from a theoretical point of view and compare our findings to the experiment.

In the following, we refer to the experimentally measured cw EPR spectra as “experimental spectra” (**exp**) and to the simulated spectra, which is the sum of all individual spectra based on the theoretically calculated *g*- and *A*-values, as “theoretical spectra” (**theo**). The spectra obtained through simulation and least-squares fitting to the **exp** and **theo** traces are referred to as “simulated spectra” (**sim**). Here, we will have to distinguish between “experimental simulated spectra (**exp-sim**) and “theoretical simulated spectra” (**theo-sim**). A further specification on the underlying data set of **theo** or **theo-sim** is denoted by “_XX-setYY-ZZ” (XX = vac or solv, YY = 1000 or 400, ZZ = QM/MM or vd or EC-RISM), with the underlying methods described in Section 3.

It is important to point out the difference between the **theo** and **theo-sim** spectra: the former is aimed at reproducing the physics of the real-world experiment as closely as possible by investigating each member of the solvated ensemble and integrating them using proper statistics. Hence, we are dealing *explicitly* with the solvated ensemble of molecules. The simulated spectrum is then a phenomenological representation of that spectrum that introduces strain parameters and effective SH parameters in order to *implicitly* account for the fact that we are dealing with a complex ensemble.

Clearly, the **theo** and **theo-sim** spectra will only coincide if the phenomenological model captures all the important effects of the ensemble in its parameterization. Deviations between the two sets of spectra indicate missing physics in phenomenological modeling.

The other interesting and meaningful comparison is the one between the **theo** and **exp** spectra. Deviations of these spectra from each other point to deficiencies in the microscopic modeling in the theoretical treatment. These can come from deficiencies in the electronic structure treatment, deficiencies in the sampling, or the molecular dynamics treatment itself. Understanding these deviations as cleanly as possible is one important objective of this work. We will come back to this essential subject in the discussion.

### 4.1 Experimental Results

**Figure 3:**
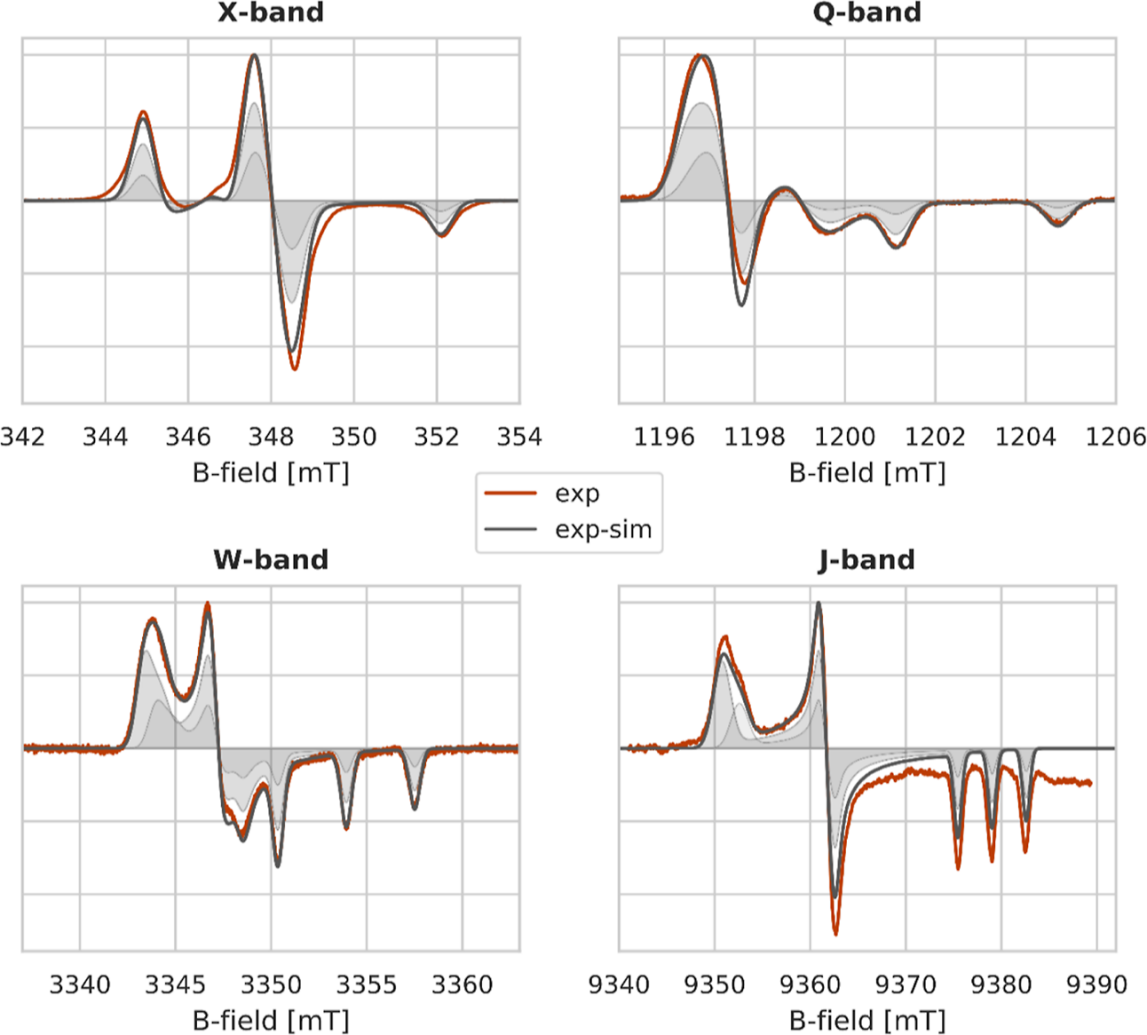
Multi-frequency cw spectra of HMI (**exp**, red line) and EasySpin-simulated spectra (**exp-sim**, gray solid line). The **exp-sim** spectra are obtained as a sum of two spectral components with a *g*_*xx*_ value of 2.00834 (***Comp1***) and 2.00795 (***Comp2***) in a 0.67:0.33 ratio (***Comp1*/*Comp2***). The two spectral components are shown as gray-filled curves, with the lighter color for ***Comp1*** and the darker color for ***Comp2***.

To analyze the effects on the EPR spectral shape of the *g*-tensor strain, a multi-frequency analysis was performed at X-, Q-, W-, and J-bands on the same sample (HMI in water at pH 10) at 100 K. The field-calibrated experimental spectra of HMI in frozen solution at four different frequencies are shown in Figure 3 (Supporting Information Section 2 and Figure S2) alongside spectral simulations obtained with eq 2.

The spectrum detected at the highest field/frequency showed the clear presence of a shoulder at the low-field region (*g*_*xx*_ region), indicating heterogeneity in the *g*_*xx*_ parameter. The main peak at a low field is characterized by a higher *g*_*xx*_ value, while the shoulder at a slightly higher field has a lower *g*_*xx*_ value. In fact, the spectra were satisfactorily simulated employing a global-fitting analysis using two sets of parameters with different *g*_*xx*_ components. Figure 3 shows the comparison between the experimental (**exp**, red) and simulated (**exp-sim**, gray) spectra, together with the populations of the two spectral components exhibiting different *g*_*xx*_. The best fit was obtained with the high (2.00834) and low (2.00795) *g*_*xx*_ components in a 0.67:0.33 ratio, respectively. The difference between the two *g*_*xx*_ parameters used is ∼4*10^–4^ (400 ppm), close to the difference previously attributed to one additional H-bond toward the nitroxide.^13^ All parameters used for the fitting are reported in Table 2.

The experimental multi-frequency data highlighted a clear heterogeneity in the *g*_*xx*_ region of the spectra of HMI in the frozen state, which can be satisfactorily reconciled with the presence of two main spectral components with different *g*_*xx*_ values of the *g*-tensor.

**Figure 4:**
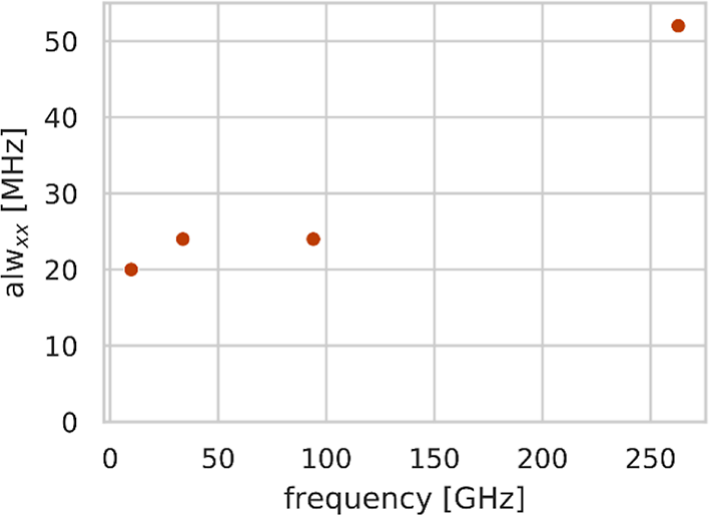
Plot of the *alw*_*xx*_ used for the *g*_*xx*_ region of the spectra vs measuring frequency, as obtained by the fits of the experimental spectra. The line width values are given in Table 2.

Furthermore, to examine the *g*-strain associated with the multi-frequency experimental spectra, we extracted orientation-dependent *alw* (*alw*_*xx*_, *alw*_*yy*_, and *alw*_*zz*_ corresponding to the line widths) as given in Table 2. The *alw* of the *g*_*yy*_- and *g*_*zz*_-signals barely vary with frequency, but the variation of the line width of the *g*_*xx*_ signal with frequency shows clearly an underlying strain beyond the W-band. In fact, while *alw*_*xx*_ changes only slightly up to W-band, a clear increase from 24 to 52 MHz is observable. The plot of *alw*_*xx*_ versus measured frequency (Figure 4) strongly resembles the example shown in Figure 1 when the field-independent *lw* dominates at low frequencies and the field-dependent effects become visible only at high frequencies. Therefore, we suggest that frequency-independent unresolved hyperfine couplings dominate the experimental spectra of HMI up to W-band, and only at the J-band does the width of the *g*_*xx*_ component increase significantly due to the *g*-strain (see the corresponding example in Figure 1).

Next, we will explore from a theoretical point of view the molecular origin of the *g*_*xx*_ heterogeneity and the *g*-strain phenomenon in terms of solvation properties, with a special focus on the role of the H-bonds created between the water molecules and the nitroxide moiety.

### 4.2 Theoretical Results I: Investigation of the Experimentally Resolved Components

#### 4.2.1 H-Bond Analysis along the AIMD Trajectory

**Figure 5:**
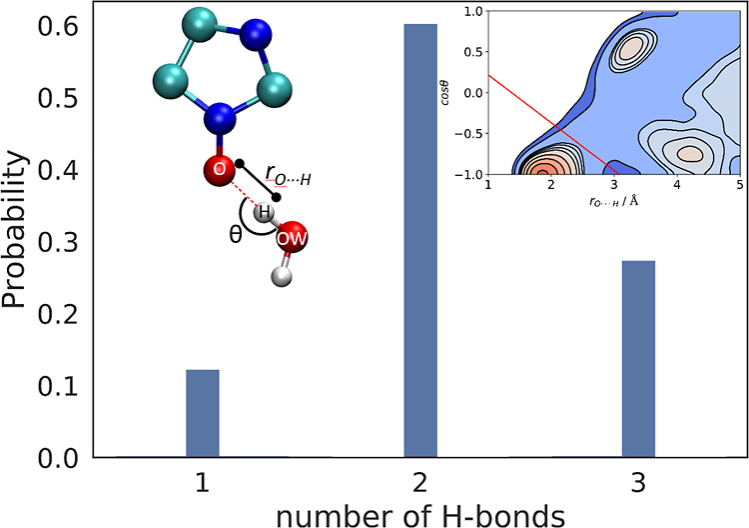
Probability distribution function of the number of HMI–water H-bonds obtained from AIMD simulations of HMI in water at ambient conditions, see text. The left inset defines the structural variables (*r*_O···H_ and cos θ) on which the employed H-bonding criterion (*r*_O···H_ < −1.71 cos θ + 1.37) is based, as explained in the text. The right inset depicts the corresponding joint probability distribution function for water molecules with respect to the oxygen site of HMI (the red line is the separatrix of H-bonded configurations located in the lower-left corner as given by the H-bonding criterion). The methyl groups of HMI in the left upper inset are not shown for the sake of clarity. As demonstrated in Supporting Information Section 4 and Figure S3, the used criterion provides essentially the same H-bond populations, including the contribution of interstitial water, as a widely used simpler criterion.

The experimental data show a heterogeneous *g*_*xx*_ region in the J-band spectrum, which indicates different subensembles of nitroxides with distinct H-bond situations. To investigate the molecular origin of this heterogeneity, we have analyzed the AIMD trajectory to extract local solvation structural information of HMI in liquid water. The population distribution function of the number of H-bonds accepted by the oxygen site of HMI is depicted in Figure 5. The preferred H-bond number is two (about 60%), followed by three (about 30%), and we found a minor contribution due to singly H-bonded HMI molecules (about 10%).

**Figure 6:**
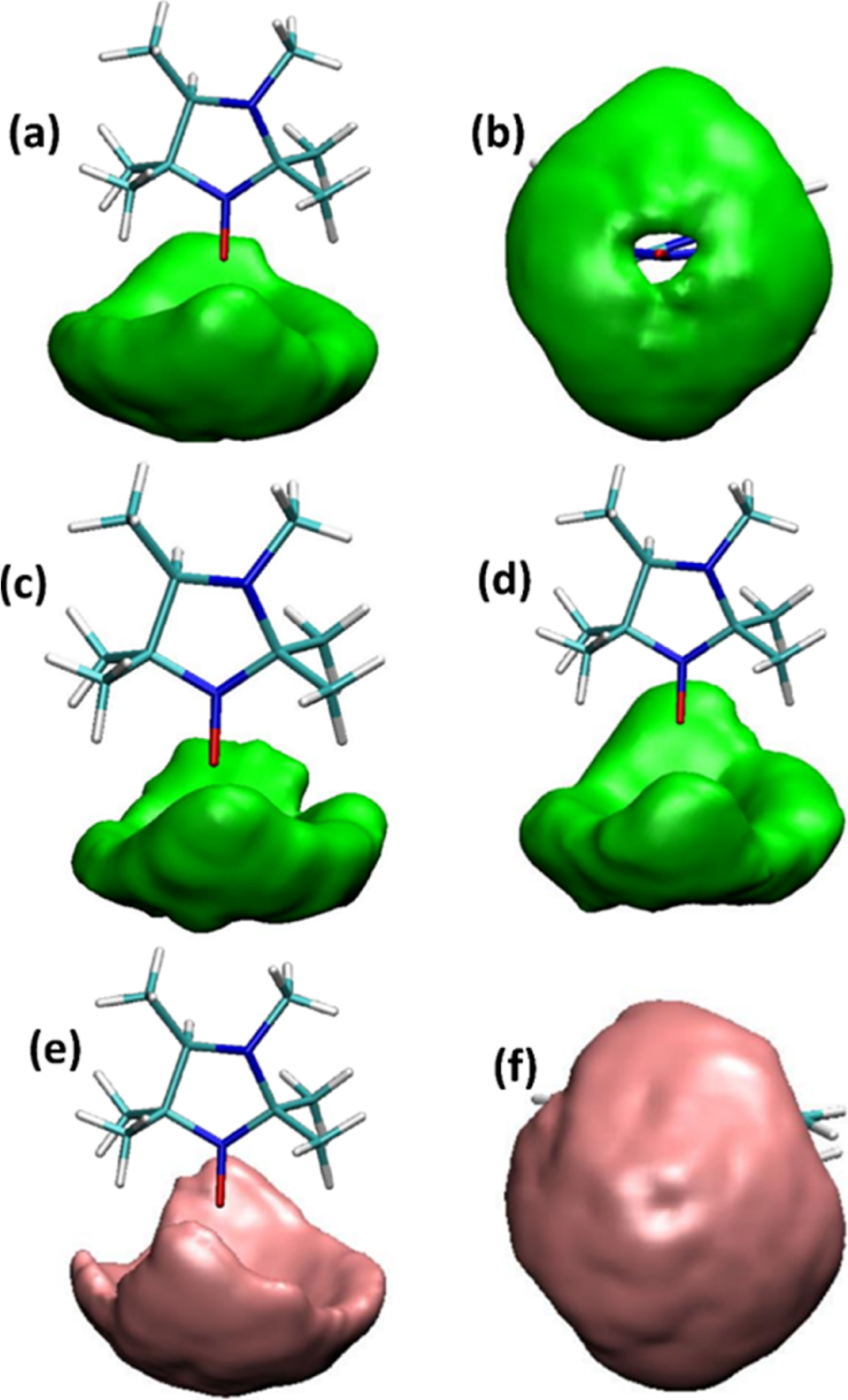
SDFs of water oxygen around the HMI oxygen for different subensembles of H-bonded and interstitial water molecules. SDFs for HMI with two H-bonds in two different orientations (a,b), with one (c), and with three (d) H-bonds. SDF in two different orientations, considering only interstitial water around the HMI oxygen (e,f). Note that in (a–d), only the H-bonded water molecules are considered, whereas (e,f) exclusively include interstitial water molecules. Additionally, we have calculated the dihedral angle distribution (Supporting Information Section 5 Figure S4) between the planes containing HMI ring atoms and hydrogen in H-bonded water for different H-bond situations. The distribution is scattered over the whole range of angles, which further supports the orientational isotropy in HMI–water H-bond formation.

Given the information on the H-bonding pattern of HMI in water, we now separate the total spatial distribution function (SDF) in terms of partial SDFs due to the 1, 2, and 3 H-bonded subensembles (see Figure 6a–d) in order to unravel their real space distributions around the HMI oxygen. Interestingly, the SDFs for these subensembles are astonishingly similar (compare panels a, c, and d), all of them featuring an approximately symmetric real space arrangement (see panels a and b) of H-bonded water molecules around HMI–O irrespective of the particular H-bonding state.

In an effort to understand this puzzling finding, we analyze the SDF contribution due to the so-called interstitial water molecules, which are those that are in the first coordination sphere (of the oxygen site of HMI) with respect to OW (water–O in this case) but not H-bonded (to the HMI oxygen). Here, the first coordination shell extends up to the first minimum of the corresponding radial distribution function at 3.3 Å, as shown in Figure 5 of ref (70). The concept of interstitial water has been found to play an important role in supercooled water.^95,96^ Later, interstitial water was demonstrated to be especially useful to understand high pressure effects on the structure of bulk water,^94^ on the solvation of biomolecules in aqueous solution,^97^ and on the proton transfer mechanism.^98^ The result presented in Figure 6 is stunning in the sense that the interstitial water molecules around the oxygen site of HMI occupy on average the same regions in space relative to HMI as the H-bonded water molecules do (compare panels e to a and panels f to b). This implies that, although one can clearly identify different H-bonding states of HMI in water, its solvation shell must be a highly labile, non-rigid structural entity. This means that the mostly 2 or 3 H-bonds that are accepted by the oxygen site of HMI are continuously breaking and reforming due to thermal fluctuations. Thus, H-bonded and interstitial water molecules are dynamically interconverting in the first solvation shell around the oxygen site of HMI in bulk water.

**Figure 7:**
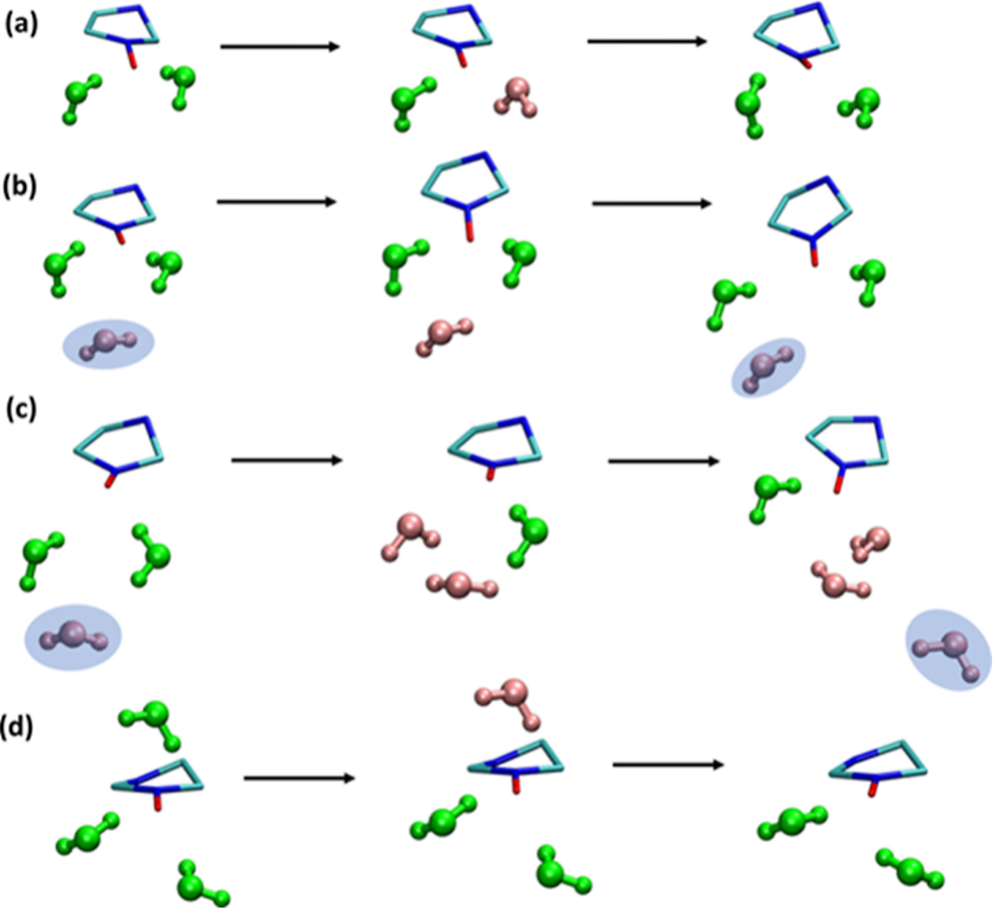
Different formation mechanisms of interstitial water molecules within the first solvation shell around the HMI oxygen site, see text for discussion. The H-bonded and interstitial water molecules are shown in green and pink, respectively. Water molecules that change their interstitial state before they enter or after they have left the first solvation shell are also included for clarity and highlighted using a blue background. The methyl groups of HMI are not shown for the sake of clarity.

We have investigated the origin of the formation of interstitial water and found mainly four mechanisms, as illustrated in Figure 7. Interstitial water can be transiently formed as a result of breaking and eventual reformation of HMI–water H-bonds within the first solvation shell due to orientational fluctuations (as depicted in panel a); it can also be generated due to translational diffusion of a water molecule from the outside into the first solvation shell either without (panel b) or with (panel c) involving existing HMI–water H-bonds; or interstitial water results from breaking H-bonds due to orientational fluctuation and eventually leaves the first solvation shell due to translational diffusion. Since the formation of interstitial water molecules has obviously no directionality, it triggers the breaking and reformation of HMI–water H-bonds without imprinting any spatial orientational preference. Hence, the spatial distribution of solvation shell water molecules results, on average, in a rather isotropic distribution of the H-bonded water molecules, which readily explains the observations made based on Figure 7.

#### 4.2.2 Theoretically Calculated EPR Parameters

In this section, we will discuss the calculated EPR parameters and compare them to the experimentally obtained quantities in an effort to assign the two species resolved in the experimental measurements.

To this end, we divided both solvated ensembles, solv-set1000 and solv-set400, into the corresponding H-bond subensembles according to the analysis described before. Taking the larger solv-set1000 as a reference, we can evaluate whether the obtained populations of H-bond subsets, *g*- and *A*-values have converged for the smaller solv-set400. Comparing both ensembles, it is found that the population of the most occurring H-bond situations, namely, 1/2/3 H-bonds, has basically converged for solv-set400. Since the *g*-values are computed at the same level of theory for both solv-set1000 and solv-set400, it can also be observed that the mean *g*-values of the subensembles have converged for solv-set400. Therefore, the mean *A*-values computed for solv-set400 are most likely converged as well. This is important to consider when comparing the theoretically determined subensembles to the experimentally resolved components.

While *two* different components are clearly recognizable in the experimental spectra, the AIMD simulations have identified *three* distinct H-bond subensembles based on analyzing the structure of the system along the calculated trajectory. The three subensembles correspond to structures featuring 1, 2, and 3 H-bonds to the nitroxide oxygen, respectively. Given that the population of the 1 H-bond contribution is fairly small in comparison to the 2 and 3 H-bond subensembles, it is possible that the experiment simply does not resolve the 1 H-bond subensemble. We will continue the analysis under this assumption and provide further evidence for its correctness below.

Taking into account only 2 and 3 H-bonding situations, the theoretical result shows good agreement with the experiment. The theoretical ratio of the H-bond populations (2 H-bond/3 H-bond) is 0.72:0.28/0.69:0.31 (solv-set400/solv-set1000), while the experimental ratio of the two observed populations is 0.67:0.33 (see Table 2). Together with the calculated SH parameters discussed below, this indicates that the experimentally resolved components can be assigned to 2 and 3 H-bonding situations, with 2 H-bonds being the most frequent. This is supported by other investigations that observe mainly two H-bonds being formed between nitroxides and solvent molecules in an aqueous solution.^22,99,100^ Nonetheless, the population of the H-bond situation as well as the population of experimentally resolved components are very sensitive to the thermodynamic conditions under which either the experiment or the MD simulations were conducted.^13^

While we have made every possible effort to ensure that theory and experiment can be properly compared, there are still some differences that should be noted and that, at least at present, cannot be avoided. These include, first and foremost, the thermodynamic conditions under which the experiments and simulations are performed and that might influence the outcome of the H-bond populations. On the one hand, the AIMD simulation temperature (300 K, see Section 3.1) was different from the experimental one (100 K, see Section 2.2), and there is no control on the speed of the samples’ freezing in liquid nitrogen in different tube sizes. On the other hand, the experimental sample contained 10% (v/v) glycerol, which is absolutely necessary as a cryoprotectant to avoid clustering of HMI in the ice. We expect only minor effects on *g*- or *A*-, supported by the finding of Hecker et al. that glycerol barely forms hydrogen bonds with nitroxides.^64^

However, sampling at the experimental thermodynamic conditions is beyond the scope of the current state-of-the-art AIMD methodology. We will therefore correlate the experimental findings with the theoretical description of the sample in a quantitative way, keeping in mind that the thermodynamic properties of the samples from which the experimental data are taken might not be fully represented by the theoretical ensemble.

The anchor point for the experimental analysis is the resolved shoulder of the *g*_*xx*_ region in the J-band measurement that leads to distinct *g*_*xx*_ values of the two observed components with a difference of Δ*g*_*xx*_ = 4*10^–4^. Unfortunately, theory at the level of revPBE0 underestimates the *g*-value for HMI. This underestimation is additionally most pronounced for larger *g*-values and therefore for the *g*_*xx*_ parameter. While the experimental analysis results in a (weighted) mean *g*_*xx*_ value of 2.00821, theory gives a mean *g*_*xx*_ value (weighted average of 1/2/3 H-bond situations, see Supporting Information Section 13, Table S2) of 2.00788. The underestimation of the *g*_*xx*_ values is independent of the solvent model since the EC-RISM calculations give a mean *g*_*xx*_ value of 2.00796 (see Table 3), which shows a similar deviation from the experiment as the calculation with explicit waters (see Supporting Information Section 7 and Figure S6 for additional comparison between AIMD and EC-RISM modeling of solvation of HMI). Therefore, we cannot rely on a comparison of the absolute *g*-values. Furthermore, neither the difference of *g*_*xx*_ values between 1 and 2 H-bonds (Δ*g*_*xx*_ = 3*10^–4^) nor 2 and 3 H-bond subensembles (Δ*g*_*xx*_ = 2*10^–4^) give quantitatively accurate values for an assignment of the experimentally resolved components. Nonetheless, the theory is able to capture the decrease of the *g*_*xx*_ value and increase of the *A*_*zz*_ value with increasing numbers of H-bonds, as observed in experimental studies.^14,15,22^

**Table 3:** Calculated EPR Parameters Based on Two Treatments of Solvation, Namely, Explicit (solv-set1000/400-QM/MM) and Implicit (solv-set1000/400-EC-RISM), in Comparison to the Experimentally Determined (Previously Given in Table 2)^a^

	**exp-sim**		solv-set1000/400-QM/MM		
	***Comp1***	***Comp2***	***TComp2*** 2H-bonds	***TComp3*** 3H-bonds	solv-set1000/400-EC-RISM
weights	0.67	0.33	0.69	0.31	
*g*_*xx*_	2.00834	2.00795	2.00791	2.00767	2.00796
(*g*_*xx*_–*g*_*zz*_)/10^–^^5^	604	565	577	553	374
*g*_*yy*_	2.00598		2.00578	2.00572	2.00576
*g*_*zz*_	2.0023		2.00214		2.0021
*A*_*xx*_ [MHz]	14		7.3/11.6	7.4/11.0	7.4/12.3
*A*_*yy*_ [MHz]	14		7.5/12.0	7.7/11.5	7.6/12.8
*A*_*zz*_ [MHz]	100		95.2/99.0	98.1/101.0	96.1/102.8

a The *g*- and *A*-values are obtained from calculations at the revPBE0 level (second *A*-value calculated at the DLPNO-CCSD level, hence based on solv-set400-QM/MM and solv-set400-EC-RISM) as the mean of the H-bond subensembles, if applicable. The weights result from the analysis of H-bonding situations along the AIMD trajectory.

However, additional insight into the comparison between theory and experiment can be derived from the computed hyperfine couplings. While the experimental difference in *g*_*xx*_ fits two different H-bond situations, only one *A*_*zz*_ value of 100 MHz could be resolved experimentally. With an experimental and theoretical (for DLPNO-CCSD^66^) error of only ±1 MHz for hyperfine coupling constants, the *A*_*zz*_ values clearly support the assignment of 2 and 3 H-bonds to the experimentally resolved components. The computed *A*_*zz*_ values barely differ at 99.0 MHz (2 H-bonds) and 101.0 MHz (3 H-bonds) and agree excellently with the experimentally derived value. By applying the EC-RISM solvation model, a mean *A*_*zz*_ value of 102.8 MHz is obtained (see Table 3), which also shows a very good agreement with the experimental reference. However, the *A*_*zz*_ value for the 1 H-bond situation (92.6 MHz) clearly deviates from the experimental value. Note that we have relied on the DLPNO-CCSD *A*-values here since those are the most accurate numbers we can achieve with the current theoretical methods at hand, as demonstrated in our previous publication.^70^ There, we have shown very good agreement between our approach to calculating the *A*_iso_ value and the experimentally measured one.

Taken together, the joint analysis of calculated populations, calculated *g*-shifts, and calculated *A*_*zz*_ values indicates that the 2 and 3 H-bond configurations should best represent the two populations identified experimentally. The detailed data of all ensembles and subensembles is given in the Supporting Information (Section 13 and Table S2), whereas a summary of the important parameters is given in Table 3.

#### 4.2.3 Overall Accuracy of Theoretically Predicted EPR Spectra

The main result of the previous section allowed us to assign the experimentally resolved components to 2 and 3 H-bond subensembles, respectively, with 2 H-bonds being the most prominent component. In this section, we will evaluate the overall accuracy of the theoretically *predicted* EPR spectrum based on the identified components.

To this end, we have simulated spectra for the 2 and 3 H-bond subensembles based on the EPR parameters given in Table 3 and subjected their weighted sum (**theo-sim**) to comparison with the experimental simulated spectrum (**exp-sim**).

**Figure 8:**
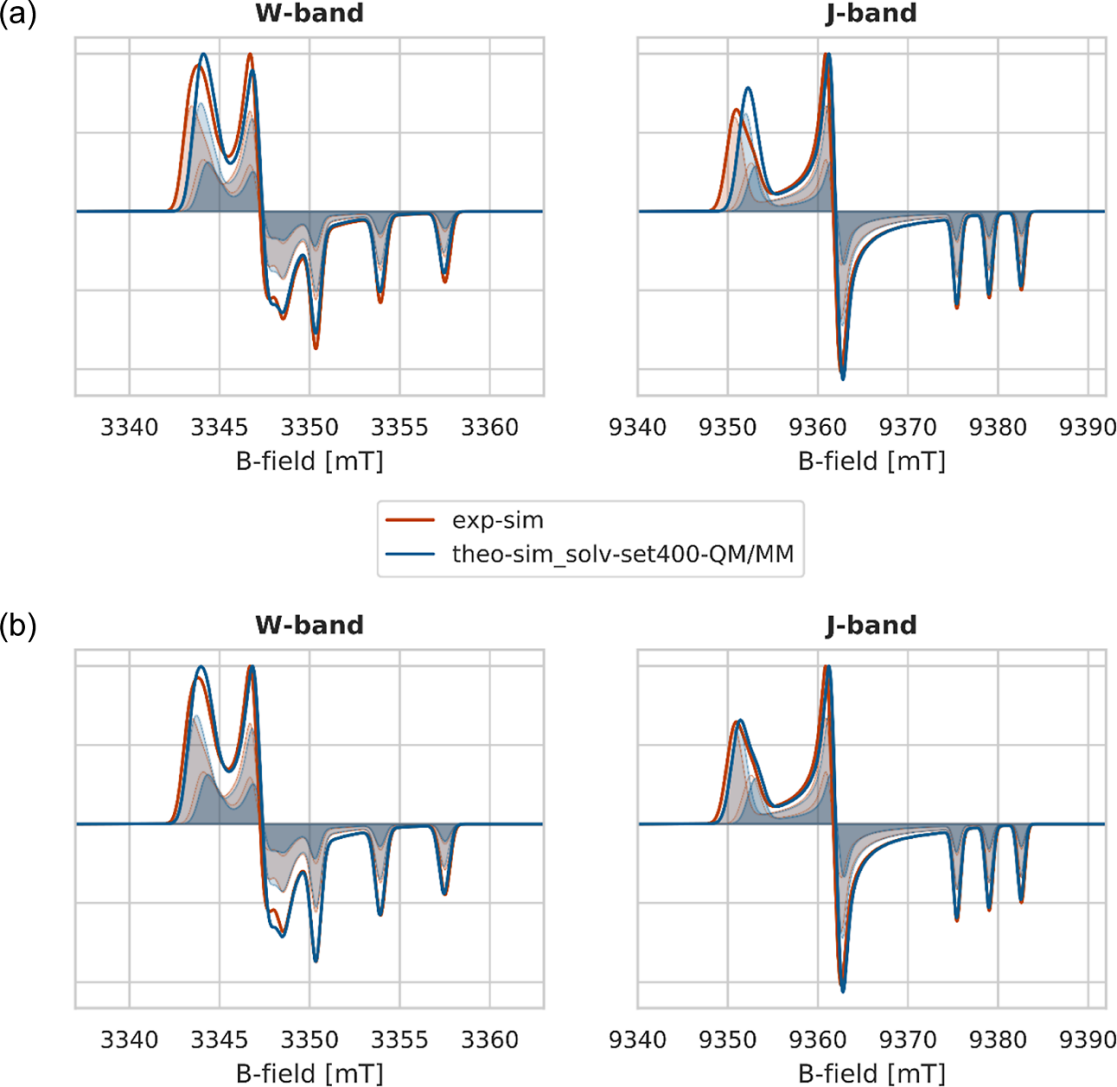
Comparison of theoretical simulated spectra (**theo-sim_solv-set400-QM/MM**, blue) based on a multicomponent Ansatz to experimental simulated spectra (**exp-sim**, red). The underlying components of the theoretical simulation are the filled blue curves. The parameters used for the simulation can be found in Table 3. Note that the DLPNO-CCSD *A*-values were used, based on the solv-set400-QM/MM data set. The theoretical spectra were shifted by 0.3 mT (W) and 0.8 mT (J), respectively, to align the experimental and theoretically simulated spectra at the *g*_*zz*_ signal. (a) All EPR parameters from calculation; line width taken from experimental analysis. (b) All EPR parameters from the calculation except for *g*_*xx*_ of ***TComp2***, which was adjusted to 2.00806 to achieve the experimental Δ*g*_*xx*_ between the spectral components, line width taken from the experimental analysis. The results obtained in the case of explicit solvation are shown in Figure S7 (Supporting Information Section 8).

**Figure 9:**
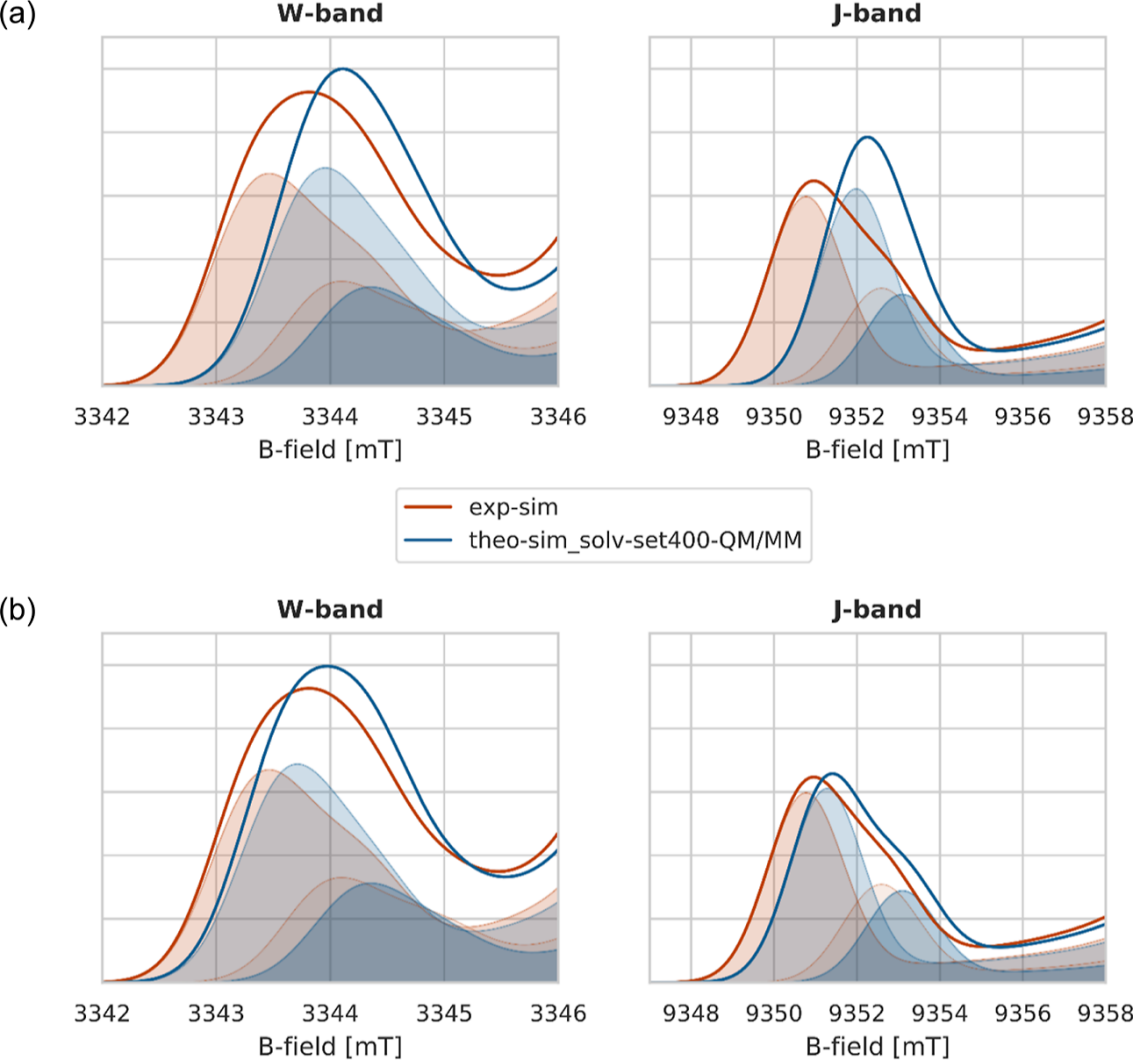
Zoom onto the *g*_*xx*_ region of the comparison between the theoretical simulated spectra (**theo-sim_solv-set400-QM/MM**, blue) based on a multicomponent Ansatz and the experimental simulated spectra (**exp-sim**, red), as fully shown in Figure 8. The results obtained in the case of explicit solvation are shown in Figure S8 of Supporting Information Section 8.

The theoretical simulated spectra (**theo-sim**, blue) are plotted alongside the experimental simulated spectra (**exp-sim**, red) in Figure 8. Here, we have focused on the W- and J-bands since they best resolve the principal *g*-values as well as the *A*_*zz*_ splitting. Note that the **theo-sim** spectra are shifted to align with the experiment at the *g*_*zz*_ signal. In Figure 8a, all EPR parameters are taken from theory, whereas the line widths were taken from the experimental analysis. The direct comparison of the **exp-sim** and **theo-sim** spectra shows very good agreement of the *A*_*zz*_ value whereas a clear underestimation of the *g*_*xx*_ value is visible in the **theo-sim** spectra. Furthermore, the experimentally resolved shoulder in the *g*_*xx*_ region is not reproduced by the **theo-sim** spectra, as visible in Figure 9. We attribute this to an underestimation of the *g*_*xx*_ differences between ***TComp2*** and ***TComp3***. Adjusting this difference by simulating ***TComp2*** with a *g*_*xx*_ value of 2.00806 instead of 2.00791 (all other parameters unchanged) yields very good agreement between theory and experiment, see Figure 9b. This shift by 150 ppm of a single parameter, which equals 2.7% of Δ*g*_*xx*_(***TComp2***) results in a visible shoulder in the *g*_*xx*_ region in the J-band spectrum. Of course, fixing the *g*_*xx*_ difference by shifting only one *g*_*xx*_ value does not overcome the overall underestimation of the *g*-values, as can be seen. Given that the deviation between theory and experiment mainly originates from the level of theory used for the *g*-tensor calculation, the agreement achieved here is nevertheless fairly satisfying.

### 4.3 Theoretical Results II—Investigation of the *g*-Strain

#### 4.3.1 Theoretical Multi-frequency cw EPR Spectra for HMI in Solution

Having accomplished a comprehensive comparison of experimental and theoretically simulated spectra, we now turn to the analysis of the line width and strain parameters. The first step to investigating the *g*-strain from a purely theoretical perspective is to generate a theoretical reference spectrum which is the normalized sum of spectra of all snapshots along the AIMD trajectory of the given ensemble. The distribution of *g*-values is the crucial quantity here, which is why we base our analysis on the larger ensemble (solv-)set1000. Comparing the theoretical spectra of solv-set1000-QM/MM and solv-set400-QM/MM shows an unconverged *g*_*xx*_ region for the smaller ensemble, as shown in Figure S9 of Supporting Information Section 9.

**Figure 10:**
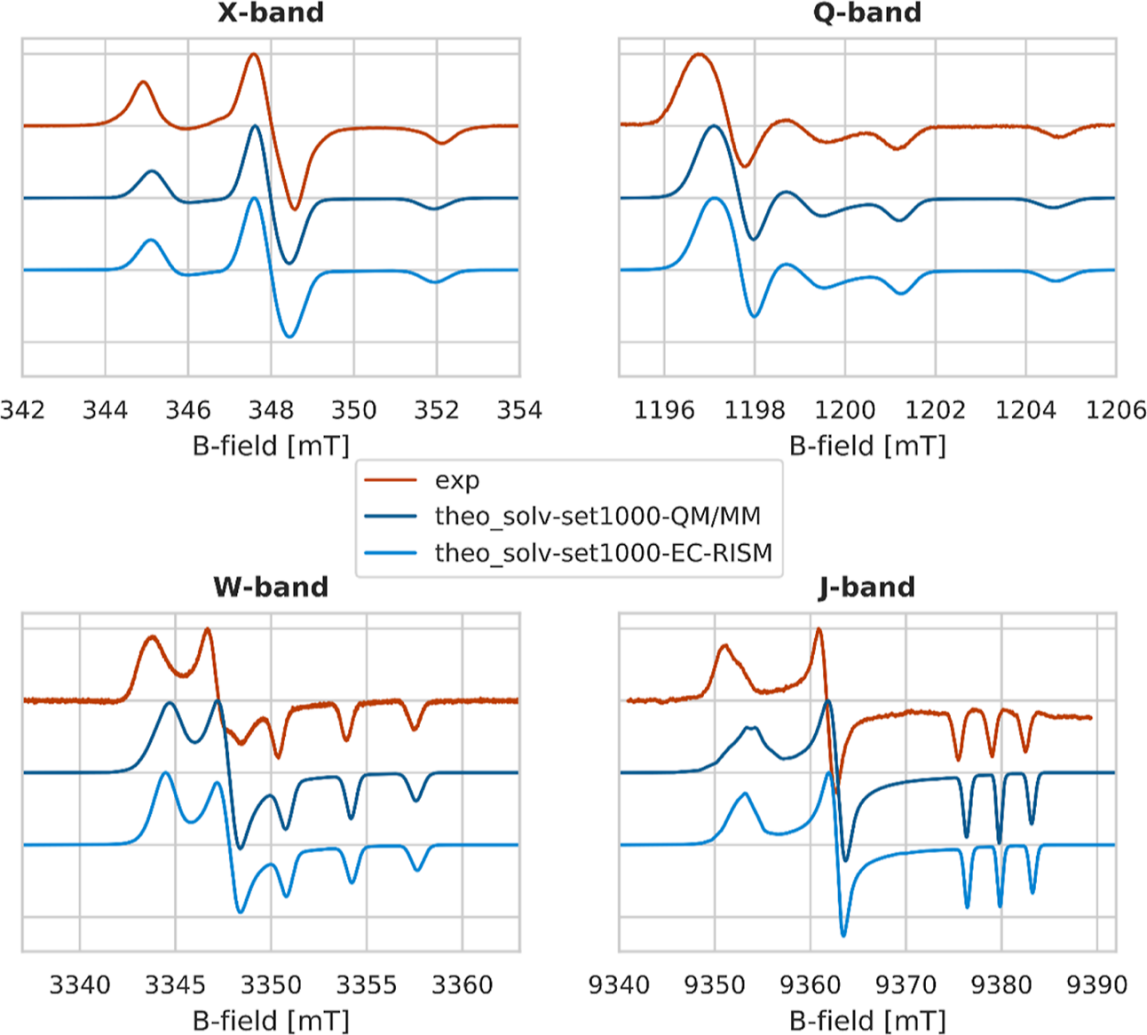
Theoretical multi-frequency spectra of HMI in water at different frequencies from computed *g*- and *A*-values based on the QM/MM approach (**theo_solv-set1000-QM/MM**) and the EC-RISM approach (**theo_solv-set1000-EC-RISM**) for the treatment of solvation plotted alongside the experimental spectra (**exp**).

The theoretical spectra (**theo_solv-set1000-QM/MM|EC-RISM**) are shown in Figure 10 alongside the experimental spectra (**exp**). Two models of the solvation environment were applied here: the QM/MM approach with explicit solvation and the EC-RISM approach. The spectra are characterized by two main interactions, hyperfine (*A*) and Zeeman (*g*), and show the typical pattern as observable for nitroxides that agrees qualitatively well with the experimental measurements. The X-band is dominated by the hyperfine interaction, whereas the Q-band depicts an intermediate regime where the Zeeman and hyperfine interactions contribute comparably. Deviations from the experimental spectra therefore occur due to an underestimation of the hyperfine interaction.

Starting from the W-band, the Zeeman interaction clearly dominates the spectrum and leads to the resolution of the principal *g*-values. Additionally, a clearly broader *g*_*xx*_ signal is observable compared to *g*_*yy*_ and *g*_*zz*_, hinting at a significant *g*-strain. The theoretical J-band improves the resolution of the *g*_*xx*_ region, which shows the start of a splitting and indicates underlying components. This agrees with the observation of the shoulder at the *g*_*xx*_ signal at the J-band measurement. While the principal *g*-values are well resolved at higher frequencies, the hyperfine interaction is never fully resolved. Deviations with respect to the experimental spectra are clearly dominated by the underestimation of the *g*-tensor components.

In addition to the problem of underestimating the *g*-tensor components due to the chosen level of theory, the extracted structures from the AIMD and the subsequent QM/MM description of the solvation can be a potential source of error. Therefore, we used EC-RISM as a second solvation model where no direct influence of the explicit waters exists because the structures were previously vertically desolvated and as a physically independent approach to account for solvation effects, as water is described within EC-RISM on a force field level (modified SPC/E water model) with thermal distribution taken from the 3D RISM approximation. Therefore, as the configurational ensembles and single-point calculation levels of theory for the QM/MM and the EC-RISM treatment are identical, the comparison of both approaches in terms of their quantitative (EPR parameter level) and qualitative (spectral shape) similarity allows us to delineate the consistency and plausibility of our characterization of solvation effects. The corresponding spectra are depicted in Figure 10. For the X-band, Q-band, and W-band, the spectra for EC-RISM and the QM/MM method look quite identical, and for both methods, the underestimation of the *g*-tensor can be observed. In the J-band spectra, the width of the EC-RISM and QM/MM spectra look visually similar, and for both methods, two shoulders (in the case of EC-RISM, at least faintly visible) can be observed. These results indicate that the solvation model does not cause deviations between the experiment and the theory.

#### 4.3.2 Simulation of the Theoretical Multi-frequency cw EPR Spectra

**Figure 11:**
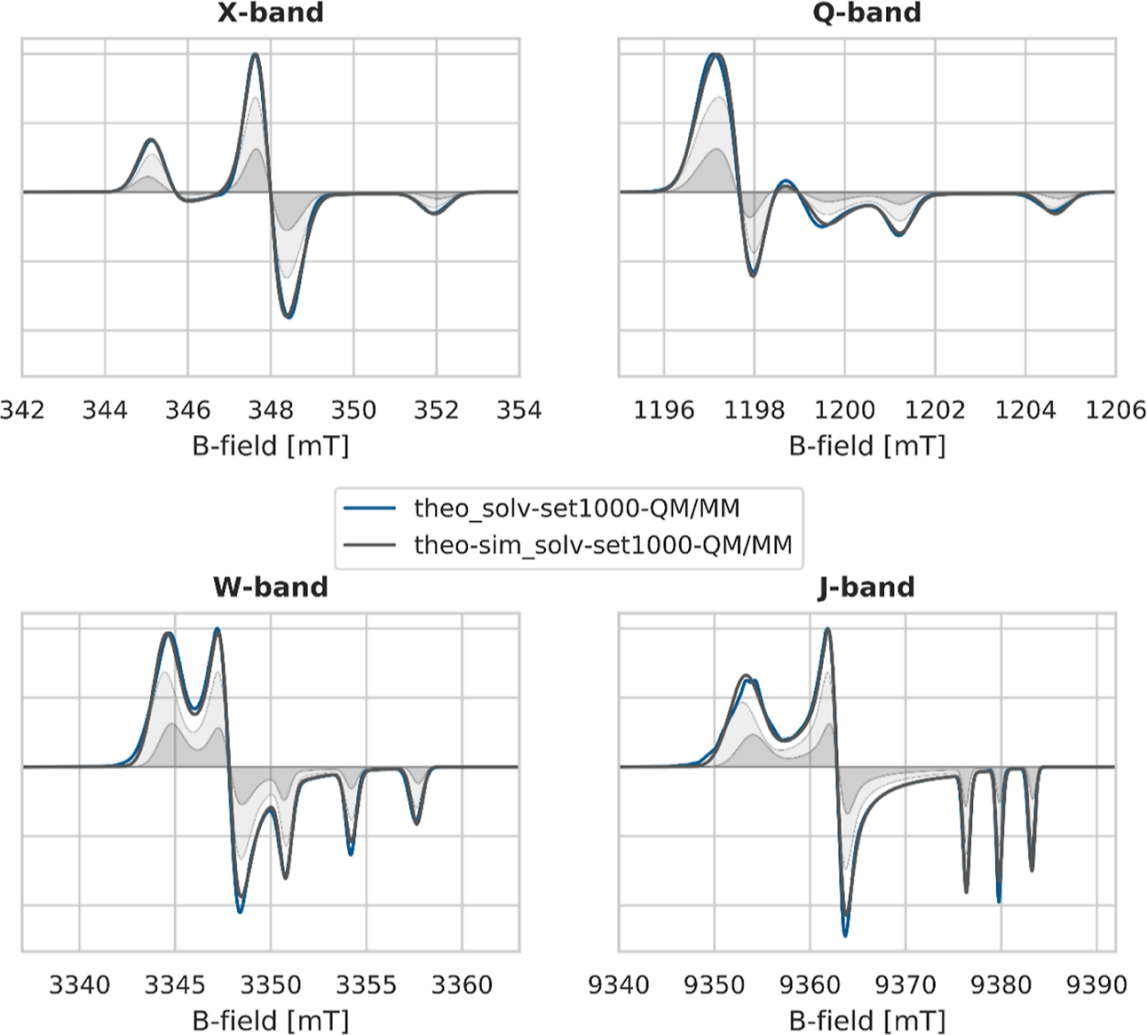
Theoretical multi-frequency cw spectra of HMI (**theo_solv-set1000-QM/MM**, blue line) and EasySpin-simulated spectra (**theo-sim_solv-set1000-QM/MM**, gray solid line). The **theo-sim_solv-set1000-QM/MM** spectra are obtained as a sum of two spectral components (gray-filled curves) in a ratio of 0.69:0.31 (***TComp2***/***TComp3***). The components and corresponding ratios are obtained from sorting the solv-set1000 AIMD trajectory into different H-bond situations around the nitroxy group, that is, ***TComp2*** = 2 H-bonds, ***TComp3*** = 3 H-bonds. The results obtained in the case of explicit solvation are shown in Figure S10 (Supporting Information Section 10).

Having established a common starting point with the theoretical spectra as a pendant to the experimental spectra, we will now quantitatively investigate the *g*-strain phenomenon from the theoretical point of view. Therefore, the theoretical multi-frequency cw EPR spectra were treated analogously to the experimental analysis based on a multicomponent Ansatz, as shown in Figure 11. Based on the assignment of the experimentally resolved spectral components from our analysis of the first part of the results, we also used two components here, which correspond to the 2 H-bonds (***TComp2***) and 3 H-bonds (***TComp3***) subensembles.

Good agreement between the theoretical simulated spectrum (**theo-sim_solv-set1000-QM/MM**) and the theoretical (**theo_solv-set1000-QM/MM**) spectrum is observed for the X-band and Q-band spectra in Figure 11. For the W and J-band spectra, the simulations deviate slightly more from each other. On the one hand, the intensities of the *g*_*yy*_ and *g*_*zz*_ regimes are not perfectly reproduced.

First, a difference in intensity pattern for the triplet peak of the *A*_*zz*_ splitting is observable in the *g*_*zz*_ region. The **theo-sim_solv-set1000-QM/MM** spectrum produces a triplet with decreasing absolute intensity, whereas the **theo_solv-set1000-QM/MM** spectrum shows a splitting pattern with the middle signal of the triplet being the most intense. This shows that the *A*_*zz*_ value varies stronger among the whole ensemble of snapshots than the simulation recovers; that is, the two different components of the simulation with their two different *A*_*zz*_ values do not capture the actual variation.

Second, the *g*_*xx*_ region is not properly reproduced by **theo-sim_solv-set1000-QM/MM**. The deviation is distinct for the J-band spectrum since the high-frequency results in a better resolution of different *g*-values. The **theo_solve-set1000-QM/MM** spectrum shows the onset of a splitting at the *g*_*xx*_ signal. Additionally, the *g*_*xx*_ signal does not show a smooth Gaussian shape. This is a clear hint of the different components underlying the total spectrum. However, those components are not well reproduced by the components of the simulation, which are classified by the number of hydrogen bonds around the nitroxy group of HMI.

Despite these deviations, an *alw* could be extracted from the simulated spectra, as given in Table 4. The line width of the *g*_*zz*_ signal is almost constant for all frequencies and components. The line width of the *g*_*yy*_ signal varies slightly with increasing frequency, therefore showing a small strain.

**Table 4:** Parameters of (**theo-sim** of the Corresponding Data Set) Used for the Fitting of the Theoretical (Multi-frequency) Spectra (**theo** of the Corresponding Data Set), Compared to the Parameters Obtained by the Fitting of the Experimental Data (Previously Presented in Table 2)^a^

		**exp-sim**^b^		**theo-sim**^c^			
				solv-set1000-QM/MM			
		***Comp1***	***Comp2***	***TComp2*** 2H-bonds	***TComp3*** 3H-bonds	solv-set1000-EC-RISM	vac-set1000
weights		0.67	0.33	0.69	0.31		
*g*_*xx*_		2.00834	2.00795	2.00791	2.00767	2.00796	2.00886
(*g*_*xx*_–*g*_*zz*_)/10^–^^5^		604	565	577	553	374	672
*g*_*yy*_		2.00598		2.00578	2.00572	2.00576	2.00604
*g*_*zz*_		2.0023		2.00214		2.0021	2.00214
*A*_*xx*_ [MHz]		14		7.3	7.4	7.4	6
*A*_*yy*_ [MHz]		14		7.5	7.7	7.6	6.3
*A*_*zz*_ [MHz]		100		95.2	98.1	96.1	83.2
*alw*_*xx*_ [MHz]	X	20		18		18	17
	Q	24		24		24	26
	W	24		40	37	35	53
	J	52		102	97	90	140
*alw*_*yy*_ [MHz]	X	20		16		17	15
	Q	22		21		20	20
	W	21		23		24	25
	J	28		45	47	40	42
*alw*_*zz*_ [MHz]	X	18		21		20	19
	Q	18		18		18	19
	W	18		19		21	19
	J	25		20		19	19

a The distinct *g*_*xx*_ components are denoted with the columns ***TComp#*** (for the theoretical spectra) and ***Comp#***. An orientation-dependent phenomenological Gaussian line broadening was considered (distinct line widths *alw*_*xx*_, *alw*_*yy*_, and *alw*_*zz*_) as specified in EasySpin with the function HStrain.

b All parameters are obtained by simulation with EasySpin of the experimental spectra.

c The *g*- and *A*-values are obtained from calculations at the revPBE0 level as the mean of the H-bond subensembles, if applicable. The weights result from the analysis of H-bond situations along the AIMD trajectory, whereas the line widths were obtained by simulation of the corresponding experimental or theoretical spectrum (previously presented for QM/MM and EC-RISM in Table 3). The vacuum results will be discussed in Section 4.3.3.

The observable behavior of the fitted *alw* for the *g*_*xx*_ signal is clearly different. A strain is observable, as already indicated by the strongly broadened *g*_*xx*_ signal in the J-band compared to the W-band. A linear correlation is observable between the *alw* and the frequency, as shown and discussed in the following in more detail, showing an underlying *g*-strain that dominates the theoretical spectra. Furthermore, the strain barely varies with the number of hydrogen bonds since the fitted *alw* for each frequency are either the same or barely vary between each component, as given in Table 4. A plot of *alw* versus frequency of ***TComp2*** and ***TComp3*** separately is given in Figure S11 (Supporting Information Section 11).

#### 4.3.3 Theoretical Multi-frequency cw EPR Spectra for HMI in Vacuum

**Figure 12:**
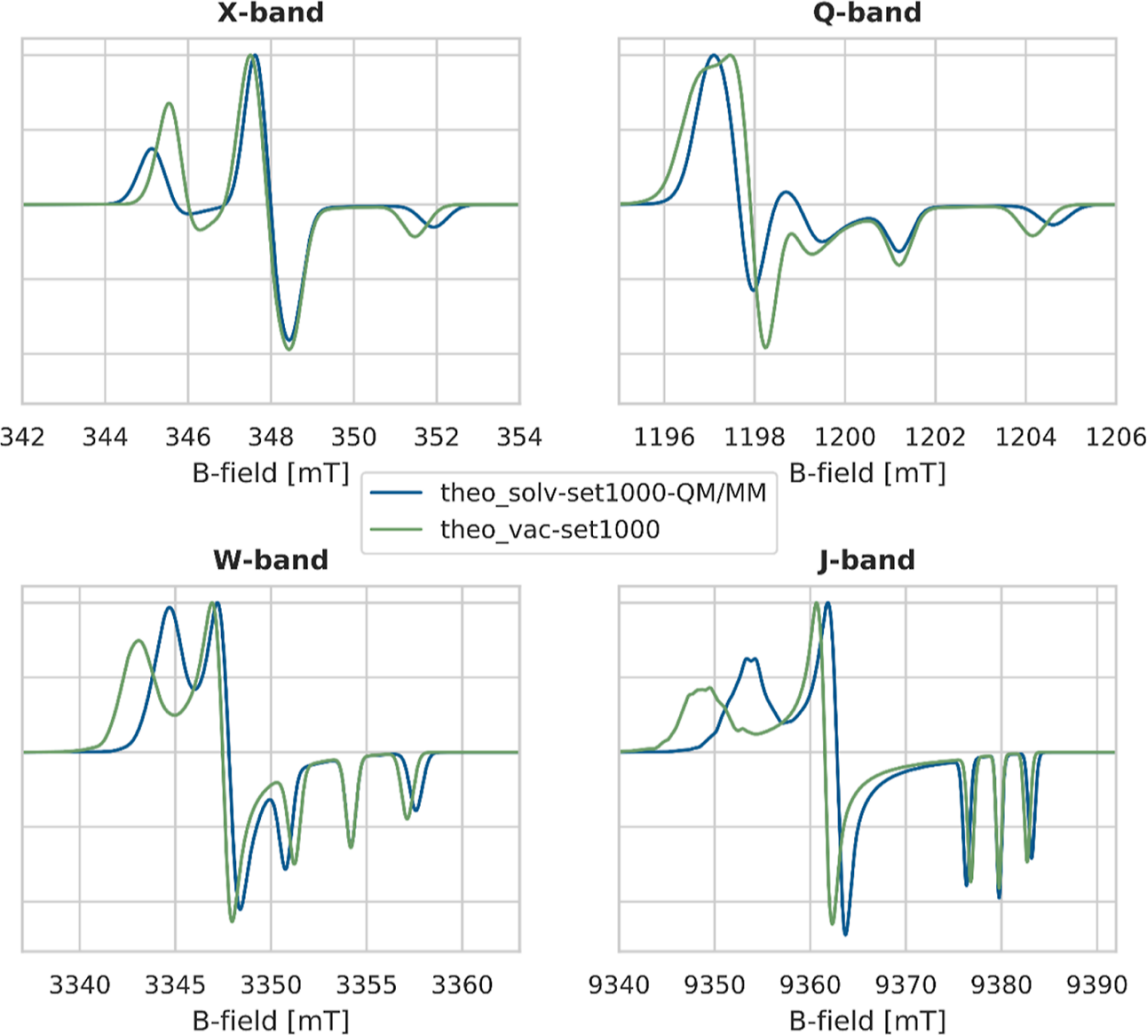
Theoretical spectra of HMI in water (**theo_solv-set1000-QM/MM**, blue) and HMI in vacuum (**theo_vac-set1000**, green) from computed *g*- and *A*-values. The final spectrum is the normalized sum of the spectra simulated for each snapshot along the corresponding AIMD trajectory.

To obtain more insight into the origin of the strain, we compared the theoretical multi-frequency spectra of solvated HMI (**theo_solv-set1000-QM/MM**) to the spectra of isolated HMI in the gas phase (**theo_vac-set1000**) that was subjected to the exact same simulation procedures as the solvated HMI considered thus far. The objective is to differentiate between strain effects that arise from the interaction of the solute with the solvent and strain effects that originate from the internal dynamics of the HMI solute. Both intermolecular interactions and internal dynamics lead to changes in SH parameters, and it is not self-evident which part is dominating over the other. Figure 12 shows the theoretical multi-frequency cw EPR spectra of HMI in water plotted alongside the theoretical spectra for HMI in vacuum.

**Figure 13:**
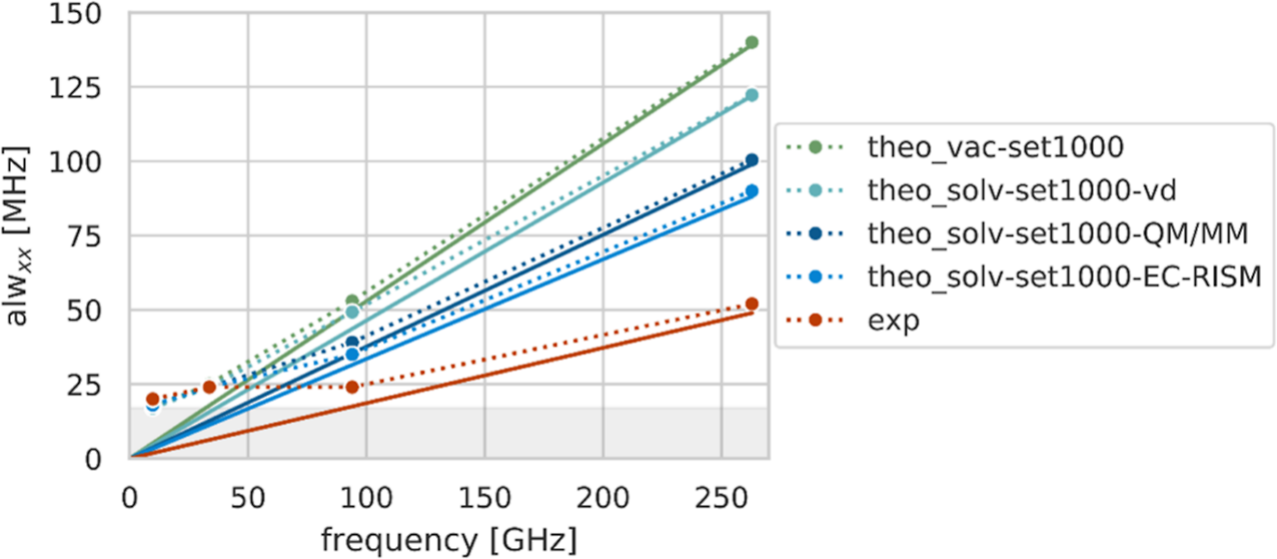
Plot of *alw*_*xx*_ vs. measuring frequency as obtained by the fits of the experimental spectra (exp), theoretical spectra of HMI in solution (theo_solv-set1000-QM/MM), vertically desolvated (theo_solv-set1000-vd), and in the gas phase (theo_vac-set1000). All theoretical spectra were created using a 17 MHz field-independent inhomogeneous line width (highlighted in gray, responsible for the plateau visible at low frequencies, see Section 3.6). The *alw* values are given in Table 4, and for “theo_solv-set1000-QM/MM” and “theo_solv-set1000-vd”, the weighted mean was taken for the W- and J-band line widths. The solid lines show a linear regression of the extracted field-dependent component of the *alw*, including the origin and the W- and J-band line widths. The *alws* (dotted lines) are dominated by the field-independent line width below 34 GHz, while at higher frequencies, the field-dependent part is more and more dominant. For the theoretical spectra, this effect is already clearly observable at 94 GHz, while in the experiment, 3 times higher frequencies are necessary to clearly identify the field dependence of the *alw*. The obtained slopes are 529 ppm (theo_vac-set1000), 464 ppm (theo_solv-set1000-vd), 376 ppm (theo_solv-set1000-QM/MM), 335 ppm (theo_solv-set1000-EC-RISM), and 186 ppm (exp) and serve as a quantification of the *g*-strain.

Besides the expected differences in the *g*- and *A*-values, a decrease of *g*_*xx*_ and an increase of *A*_*zz*_ upon solvation, Figure 12 shows that an even *broader* spectrum is obtained for the vacuum data compared to the spectrum calculated in solution. This is especially evident for the *g*_*xx*_ signal in the J-band spectra. An analysis of the *alw* of the vacuum data shows the increase of line width with frequency, which is even stronger than for the solvated data ensemble. This indicates that the *g*-strain originates from the configurational variation of the molecule itself, as visualized in Figure 13. The data for the simulation of the theoretical spectra in vacuum and the obtained *alws* are given in Table 4 next to the experimental and theoretical data in solution.

#### 4.3.4 Discussion of the Size and Origin of the *g*-Strain

Both theory and experiments indicate that the field-dependent line width originates from at least two different effects. The first effect is assigned to a varying number of hydrogen bonds in the probed molecular ensemble, and the second to the *g*-strain associated with each subensemble. The subensembles characterized by 2 and 3 H-bonds have different mean *g*_*xx*_ values, which cannot be resolved at lower fields (X, Q, and W) but can be resolved at the J-band. In fact, one *g*_*xx*_ component was not sufficient to fit the low-field region of the J-band spectra, indicating the presence of two *g*_*xx*_ subensembles. The presence of unresolved different *g*_*xx*_ values, originating from the subensembles contributes to the frequency-dependent *alw* at low fields, but its effect is small with respect to the field-independent broadening, as shown experimentally (Figure 13). Each *g*_*xx*_ subensemble is characterized by its own intrinsic *g*-strain, which cannot be further resolved into distinct heterogeneous components at the J-band. Our data analysis demonstrates that the *g*-strain associated with each subensemble is the same (see Table 3). This additional *g*-strain effect is the extracted field-dependent *alw* presented in Figure 13 and will be discussed in the following.

The values of the slope obtained from a linear regression of the extracted field-dependent contribution of the fitted *alw* (computed according to eq 1) from the experimental (186 ppm, exp, Figure 13) and theoretical spectra of HMI in solution (376 ppm, theo_solv-set1000-QM/MM, Figure 13) clearly demonstrate that theory overestimates the line width by roughly a factor of two. Analysis of the spectral components assigned to different numbers of hydrogen bonds does not explain this broadening. On the contrary, as we have shown above, the splitting of the *g*_xx_ component due to different hydrogen bonds is smaller than the shifts experimentally determined in the experiment (theo_solv-set1000-QM/MM: 200 ppm, exp: 400 ppm). Hence, the inhomogeneous line broadening is dominated by the distinct *g*_*xx*_ values of the spectral components (***Comp1*** and ***Comp2***, as clearly resolved at J-band) in the experiment, while the solvated theoretical spectra are clearly dominated by the *g*-strain.

Despite the overestimation of the strain by theory, it is highly instructive to dissect the effects arising from the solvation shell and from the structural distortion of the HMI itself. The effect of the solvation and H-bonding on the *g*-strain can be inferred by comparing the *g*-strain in the theoretical spectra of HMI in solution (theo_solv-set1000-QM/MM) with that obtained in the vertically desolvated (theo_solv-set1000-vd) HMI set, in which all water molecules (including the H-bonds) were removed, but the configurations of the HMI are those induced by the explicit solvent. To obtain insights into the effects of the structural deformation of the HMI molecule itself, we can analyze the *g*-strain obtained in the gas phase (theo_vac-set1000) sets, in which no solvent molecules are present.

It is probably fair to state that most EPR spectroscopists would intuitively ascribe the bulk of the strain to heterogeneous intermolecular interactions (HMI with solvent molecules) over the ensemble of molecules in a frozen solution.^17,101^ However, this is not what is found in our calculations. First, the theoretical data indicate that the increase of *alw* with respect to spectrometer frequency is nearly identical for spectral components originating from a given number of hydrogen bonds (see Supporting Information Section 11 and Figure S11), confirming previous observations.^15^

The EC-RISM calculations support this finding. In fact, a similarly overestimated *g*-strain (335 ppm, see Figure 13) is observed as for the QM/MM calculations, though a conceptually different solvation model was used. Since no explicit water molecules are considered in the EC-RISM calculations, and therefore, no distinction between different H-bond situations can be made, this shows the independence of the *g*-strain from the number of hydrogen bonds.

Second, when HMI is desolvated in silico (theo_solv-set1000-vd), the *g*-strain effects are even larger with a slope of 464 ppm than those obtained with the fully solvated set. Therefore, we can conclude that the solvation environment of HMI (including the H-bonding networks), despite being highly heterogeneous, overall decreases the *g*-strain. This is further supported by the analysis of the 2 and 3 H-bond subensembles, which were partially vertically desolvated, that is keeping HMI and the solvent molecules that form H-bonds toward the nitroxy group. These additional data named “theo_solv-set1000-hb2vd” and “theo_solv-set1000-hb3vd” follow the trend observed, thus positioning themselves in between theo_solv-set1000-QM/MM and theo_solv-set1000-vd. In fact, these vertically desolvated H-bond clusters lie very close to theo_solv-set1000-QM/MM and therefore show that the explicit formation of H-bonds alone contributes distinctly to the decrease of the *g*-strain in addition to the indirect effects of solvation on the electronic structure, as shown by theo_solv-set1000-vd. Additional data and a more detailed comparison are given in the Supporting Information (Section 11, Figures S12, S13, and Table S1).

Most interestingly, the HMI in vacuum shows that the largest amount of *g*-strain (529 ppm) already exists in the unsolvated system (theo_vac-set1000) and therefore originates from the configurational flexibility of the nitroxide itself (see Supporting Information Section 12 Figure S14, where it is shown that the configurational flexibility of HMI is very similar in the liquid and gas phases at the same temperature of 300 K).

This indicates that, at least in the case of HMI and in the way we have theoretically treated it, the *intra*molecular strain component dominates over the *inter*molecular strain for a given H-bonded subensemble (e.g., 2 H-bonded HMI or 3 H-bonded HMI). However, as clearly visible by the experimentally resolved subensembles’ spectral fractions at J-band, the net variation of *g*_*xx*_ (400 ppm) due to the presence of 2 or 3 H-bonds exceeds the *g*-strain of each individual subensemble (186 ppm).

Interestingly, even though the effective number of hydrogen bonds in a subensemble does not affect the strain, hydrogen bonding, or more generally, solvation of the nitroxide molecule, has a theoretically calculated effect. The spectra become *narrower* upon solvation of the HMI molecule, and the apparent line width increases with frequency; that is, a direct measurement for the *g*-strain decreases, as is clearly shown in Figure 13. This is in line with the experimental finding that radical cofactors in confined protein binding pockets, which may potentially restrict structural flexibility, show strongly reduced *g*-strain as compared to the same radicals in frozen solutions.^20,21^

## 5 Conclusions

In this study, we extended our approach of combining state-of-the-art theoretical methodologies to compute accurate EPR parameters, namely, *g*- and *A*-values, of the nitroxide HMI in aqueous solution. This allowed a direct comparison of the simulated EPR spectra with the experimental data. By analyzing the AIMD trajectory with respect to the occurring H-bonds, we could divide our set of *g*- and *A*-values into subsets of different H-bond subensembles. The mean *g*_*xx*_ and *A*_*zz*_ values of the subensembles show the expected correlation for nitroxides, that is, *g*_*xx*_ decreases and *A*_*zz*_ increases upon increasing numbers of H-bonds. Furthermore, the H-bond analysis allowed us to associate the underlying experimental spectral components with nitroxide moieties characterized by 2 and 3 H-bonds. The simulated spectra based on the theoretical components are in good agreement with the experiments. The *A*_*zz*_ value agrees with the experimental one within the error margin of ±1 MHz, whereas the *g*-values are slightly underestimated, especially the largest *g*_*xx*_ value, which corresponds to the prevalent 2 H-bond component.

To take a step further, we then investigated the *g*-strain from a purely theoretical approach at the highest realistically affordable level of detail. Comparing our results to the experimental measurements reveals that the theory strongly overestimates the *g*-strain. Interestingly, our results imply that the *g*-strain is barely affected by the actual number of H-bonds in each subensemble but rather originates from the configurational fluctuation of the molecule itself as a strain is already observable in the analysis in vacuum. This is additionally supported by the experimental finding that one set of line width parameters is sufficient for both *g*_*xx*_ components. Although the number of H-bonds does not affect the *g*-strain, a change in the solvation environment does. The calculations predict that the *g*-strain *decreases* upon solvation, presumably due to the restricted degrees of freedom of the molecule itself caused by the interaction with the solvent molecules. Maybe slightly counterintuitively, this is nonetheless supported by experimental findings that radical cofactors in confined protein binding pockets exhibit a strongly reduced *g*-strain as compared to the same radicals in frozen solutions.^20,21^

## Supporting Information Available

Experimental spectra, magnetic field calibration methods, H-bond criterion, dihedral angle distributions, solvation modeling, theoretical simulated spectra at different levels of theory, theoretical line width analysis, structural properties of HMI in gas vs liquid phase, and summary of all theoretical data (PDF)

## References

[ref1] The Royal Society of Chemistry. Nitroxides; The Royal Society of Chemistry, 2021.

[ref2] Haywood R. Spin-Trapping: Theory and Applications. In Encyclopedia of Biophysics; Roberts G. C. K., Ed.; Springer Berlin Heidelberg: Berlin, Heidelberg, 2013; pp 2447–2453.

[ref3] Jeschke G. Dipolar Spectroscopy – Double-Resonance Methods. In eMagRes; Wiley, 2013; pp 1459–1476.

[ref4] Berliner J. L.; Eaton G. R.; Eaton S. S. Distance Measurements in Biological Systems by EPR; Springer: New York, NY, 2012.

[ref5] Hemminga M.; Berliner L. ESR Spectroscopy in Membrane Biophysics; Springer Science & Business Media, 2007.

[ref6] Berliner L. J.; Reuben J. Spin Labeling; Springer: New York, NY, 2011.

[ref7] Berliner L. J. Spin Labeling. The Next Millennium; Springer: New York, NY, 2020.

[ref8] Bordignon E. EPR Spectroscopy of Nitroxide Spin Probes; John Wiley & Sons, Ltd., 2017; Vol. 6.

[ref9] Plato M.; Steinhoff H.-J.; Wegener C.; Törring J. T.; Savitsky A.; Möbius K. Molecular orbital study of polarity and hydrogen bonding effects on the g and hyperfine tensors of site directed NO spin labelled bacteriorhodopsin. Mol. Phys. 2002, 100, 3711–3721. 10.1080/00268970210166246.

[ref10] Owenius R.; Engström M.; Lindgren M.; Huber M. Influence of Solvent Polarity and Hydrogen Bonding on the EPR Parameters of a Nitroxide Spin Label Studied by 9-GHz and 95-GHz EPR Spectroscopy and DFT Calculations. J. Phys. Chem. A 2001, 105, 10967–10977. 10.1021/jp0116914.

[ref11] Bordignon E.; Brutlach H.; Urban L.; Hideg K.; Savitsky A.; Schnegg A.; Gast P.; Engelhard M.; Groenen E. J. J.; Möbius K.; Steinhoff H.-J. Heterogeneity in the Nitroxide Micro-Environment: Polarity and Proticity Effects in Spin-Labeled Proteins Studied by Multi-Frequency EPR. Appl. Magn. Reson. 2010, 37, 391–403. 10.1007/s00723-009-0072-9.

[ref12] Haugland M. M.; Anderson E. A.; Lovett J. E. Tuning the properties of nitroxide spin labels for use in electron paramagnetic resonance spectroscopy through chemical modification of the nitroxide framework. In Electron Paramagnetic Resonance: Volume 25; Chechik V., Murphy D. M., Eds.; The Royal Society of Chemistry, 2016.

[ref13] Bordignon E.; Nalepa A. I.; Savitsky A.; Braun L.; Jeschke G. Changes in the Microenvironment of Nitroxide Radicals around the Glass Transition Temperature. J. Phys. Chem. B 2015, 119, 13797–13806. 10.1021/acs.jpcb.5b04104. 26266707

[ref14] Gast P.; Herbonnet R. T. L.; Klare J.; Nalepa A.; Rickert C.; Stellinga D.; Urban L.; Möbius K.; Savitsky A.; Steinhoff H.-J.; Groenen E. J. J. Hydrogen bonding of nitroxide spin labels in membrane proteins. Phys. Chem. Chem. Phys. 2014, 16, 15910–15916. 10.1039/c4cp01741b. 24964099

[ref15] Nalepa A.; Möbius K.; Plato M.; Lubitz W.; Savitsky A. Nitroxide Spin Labels-Magnetic Parameters and Hydrogen-Bond Formation: A High-Field EPR and EDNMR Study. Appl. Magn. Reson. 2019, 50, 1–16. 10.1007/s00723-018-1073-3.

[ref16] Jeschke G.; Polyhach Y. Distance measurements on spin-labelled biomacromolecules by pulsed electron paramagnetic resonance. Phys. Chem. Chem. Phys. 2007, 9, 1895–1910. 10.1039/b614920k. 17431518

[ref17] Hagen W. R., EPR spectroscopy of Iron-Sulfur proteins. In Advances in Inorganic Chemistry; Academic Press, 1992; Vol. 38; pp 165–222.

[ref18] Hagen W. R.; Hearshen D. O.; Sands R. H.; Dunham W. R. A statistical theory for powder EPR in distributed systems. J. Magn. Reson. 1985, 61, 220–232. 10.1016/0022-2364(85)90077-0.

[ref19] Pilbrow J. R. Lineshapes in frequency-swept and field-swept epr for spin. J. Magn. Reson. 1984, 58, 186–203. 10.1016/0022-2364(84)90209-9.

[ref20] Duboc-Toia C.; Hassan A. K.; Mulliez E.; Ollagnier-de Choudens S.; Fontecave M.; Leutwein C.; Heider J. Very High-Field EPR Study of Glycyl Radical Enzymes. J. Am. Chem. Soc. 2003, 125, 38–39. 10.1021/ja026690j. 12515500

[ref21] Bratt P. J.; Ringus E.; Hassan A.; Tol H. V.; Maniero A.-L.; Brunel L.-C.; Rohrer M.; Bubenzer-Hange C.; Scheer H.; Angerhofer A. EPR on Biological Samples beyond the Limits of Superconducting MagnetsThe Primary Donor Cation of Purple Bacterial Photosynthesis. J. Phys. Chem. B 1999, 103, 10973–10977. 10.1021/jp992885a.

[ref22] Savitsky A.; Nalepa A.; Petrenko T.; Plato M.; Möbius K.; Lubitz W. Hydrogen-Bonded Complexes of Neutral Nitroxide Radicals with 2-Propanol Studied by Multifrequency EPR/ENDOR. Appl. Magn. Reson. 2022, 53, 1239–1263. 10.1007/s00723-021-01442-y.

[ref23] Hagen W. R. Biomolecular EPR Spectroscopy; CRC Press, Taylor & Francis Group: Boca Raton London New York, 2009; p 249.

[ref24] Giovannini T.; Cappelli C. Continuum vs. atomistic approaches to computational spectroscopy of solvated systems. Chem. Commun. 2023, 59, 5644–5660. 10.1039/d2cc07079k. 37074209

[ref25] Barone V.; Puzzarini C.; Mancini G. Integration of theory, simulation, artificial intelligence and virtual reality: a four-pillar approach for reconciling accuracy and interpretability in computational spectroscopy. Phys. Chem. Chem. Phys. 2021, 23, 17079–17096. 10.1039/d1cp02507d. 34346437

[ref26] Barone V.; Alessandrini S.; Biczysko M.; Cheeseman J. R.; Clary D. C.; McCoy A. B.; DiRisio R. J.; Neese F.; Melosso M.; Puzzarini C. Computational molecular spectroscopy. Nat. Rev. Methods Primers 2021, 1, 38 10.1038/s43586-021-00034-1.

[ref27] Giovannini T.; Egidi F.; Cappelli C. Molecular spectroscopy of aqueous solutions: a theoretical perspective. Chem. Soc. Rev. 2020, 49, 5664–5677. 10.1039/c9cs00464e. 32744278

[ref28] Giovannini T.; Marrazzini G.; Scavino M.; Koch H.; Cappelli C. Integrated Multiscale Multilevel Approach to Open Shell Molecular Systems. J. Chem. Theory Comput. 2023, 19, 1446–1456. 10.1021/acs.jctc.2c00805. 36780359 PMC10018740

[ref29] Neese F. Quantum chemistry and EPR parameters. In eMagRes; John Wiley & Sons, Ltd: Chichester, UK, 2017; Vol. 6, pp 1–22.

[ref30] Munzarová M. L. DFT Calculations of EPR Hyperfine Coupling Tensors. In Calculation of NMR and EPR Parameters; Kaupp M., Bühl M., Malkin V. G., Eds.; Wiley-VCH Verlag GmbH & Co. KGaA: Weinheim, FRG, 2004; pp 461–482.

[ref31] Kaupp M. Ab Initio and Density Functional Calculations of Electronic g-Tensors for Organic Radicals. In EPR of Free Radicals in Solids I; Lund A., Shiotani M., Eds.; Springer Netherlands: Dordrecht, 2013, Vol. 24, pp 323–361.

[ref32] Kaupp M. Calculation of NMR and EPR Parameters: Theory and Applications, 1 ed.; Wiley, 2004.

[ref33] Falbo E.; Fuse M.; Lazzari F.; Mancini G.; Barone V. Integration of Quantum Chemistry, Statistical Mechanics, and Artificial Intelligence for Computational Spectroscopy: The UV-Vis Spectrum of TEMPO Radical in Different Solvents. J. Chem. Theory Comput. 2022, 18, 6203–6216. 10.1021/acs.jctc.2c00654. 36166322 PMC9558374

[ref34] Schulz C. E.; van Gastel M.; Pantazis D. A.; Neese F. Converged Structural and Spectroscopic Properties for Refined QM/MM Models of Azurin. Inorg. Chem. 2021, 60, 7399–7412. 10.1021/acs.inorgchem.1c00640. 33939922 PMC8154437

[ref35] Schulz C. E.; Castillo R. G.; Pantazis D. A.; DeBeer S.; Neese F. Structure-Spectroscopy Correlations for Intermediate Q of Soluble Methane Monooxygenase: Insights from QM/MM Calculations. J. Am. Chem. Soc. 2021, 143, 6560–6577. 10.1021/jacs.1c01180. 33884874 PMC8154522

[ref36] Egidi F.; Angelico S.; Lafiosca P.; Giovannini T.; Cappelli C. A polarizable three-layer frozen density embedding/molecular mechanics approach. J. Chem. Phys. 2021, 154, 164107 10.1063/5.0045574. 33940798

[ref37] Vogler S.; Dietschreit J. C. B.; Peters L. D. M.; Ochsenfeld C. Important components for accurate hyperfine coupling constants: electron correlation, dynamic contributions, and solvation effects. Mol. Phys. 2020, 118, e1772515 10.1080/00268976.2020.1772515.

[ref38] Reimann M.; Kaupp M. Evaluation of an Efficient 3D-RISM-SCF Implementation as a Tool for Computational Spectroscopy in Solution. J. Phys. Chem. A 2020, 124, 7439–7452. 10.1021/acs.jpca.0c06322. 32838530

[ref39] Giovannini T.; Lafiosca P.; Chandramouli B.; Barone V.; Cappelli C. Effective yet reliable computation of hyperfine coupling constants in solution by a QM/MM approach: Interplay between electrostatics and non-electrostatic effects. J. Chem. Phys. 2019, 150, 124102 10.1063/1.5080810. 30927869

[ref40] Schattenberg C. J.; Kaupp M. Extended Benchmark Set of Main-Group Nuclear Shielding Constants and NMR Chemical Shifts and Its Use to Evaluate Modern DFT Methods. J. Chem. Theory Comput. 2021, 17, 7602–7621. 10.1021/acs.jctc.1c00919. 34797677

[ref41] Schattenberg C. J.; Kaupp M. Effect of the Current Dependence of Tau-Dependent Exchange-Correlation Functionals on Nuclear Shielding Calculations. J. Chem. Theory Comput. 2021, 17, 1469–1479. 10.1021/acs.jctc.0c01223. 33629849

[ref42] Schattenberg C. J.; Kaupp M. Implementation and Validation of Local Hybrid Functionals with Calibrated Exchange-Energy Densities for Nuclear Shielding Constants. J. Phys. Chem. A 2021, 125, 2697–2707. 10.1021/acs.jpca.1c01135. 33730855

[ref43] Tran V. A.; Neese F. Double-hybrid density functional theory for g-tensor calculations using gauge including atomic orbitals. J. Chem. Phys. 2020, 153, 054105 10.1063/5.0013799. 32770923

[ref44] Ghassemi Tabrizi S.; Arbuznikov A. V.; Jimenez-Hoyos C. A.; Kaupp M. Hyperfine-Coupling Tensors from Projected Hartree-Fock Theory. J. Chem. Theory Comput. 2020, 16, 6222–6235. 10.1021/acs.jctc.0c00617. 32841008

[ref45] Schattenberg C. J.; Reiter K.; Weigend F.; Kaupp M. An Efficient Coupled-Perturbed KohnSham Implementation of NMR Chemical Shift Computations with Local Hybrid Functionals and Gauge-Including Atomic Orbitals. J. Chem. Theory Comput. 2020, 16, 931–943. 10.1021/acs.jctc.9b00944. 31899647

[ref46] Dittmer A.; Stoychev G. L.; Maganas D.; Auer A. A.; Neese F. Computation of NMR Shielding Constants for Solids Using an Embedded Cluster Approach with DFT, Double-Hybrid DFT, and MP2. J. Chem. Theory Comput. 2020, 16, 6950–6967. 10.1021/acs.jctc.0c00067. 32966067 PMC7659039

[ref47] Barone V.; Fuse M. Accurate Structures and Spectroscopic Parameters of Phenylalanine and Tyrosine in the Gas Phase: A Joint Venture of DFT and Composite Wave-Function Methods. J. Phys. Chem. A 2023, 127, 3648–3657. 10.1021/acs.jpca.3c01174. 37052318 PMC10150396

[ref48] Bangerter F. H.; Glasbrenner M.; Ochsenfeld C. Tensor-Hypercontracted MP2 First Derivatives: Runtime and Memory Efficient Computation of Hyperfine Coupling Constants. J. Chem. Theory Comput. 2022, 18, 5233–5245. 10.1021/acs.jctc.2c00118. 35943450 PMC9476664

[ref49] Walisinghe A. J.; Chilton N. F. Assessment of minimal active space CASSCF-SO methods for calculation of atomic Slater-Condon and spin-orbit coupling parameters in d- and f-block ions. Dalton Trans. 2021, 50, 14130–14138. 10.1039/d1dt02346b. 34623369 PMC9583075

[ref50] Auer A. A.; Tran V. A.; Sharma B.; Stoychev G. L.; Marx D.; Neese F. A case study of density functional theory and domain-based local pair natural orbital coupled cluster for vibrational effects on EPR hyperfine coupling constants: vibrational perturbation theory versus ab initio molecular dynamics. Mol. Phys. 2020, 118, e1797916–e1797920. 10.1080/00268976.2020.1797916.

[ref51] Stoychev G. L.; Auer A. A.; Gauss J.; Neese F. DLPNO-MP2 second derivatives for the computation of polarizabilities and NMR shieldings. J. Chem. Phys. 2021, 154, 164110 10.1063/5.0047125. 33940835

[ref52] Birnoschi L.; Chilton N. F. Hyperion: A New Computational Tool for Relativistic Ab Initio Hyperfine Coupling. J. Chem. Theory Comput. 2022, 18, 4719–4732. 10.1021/acs.jctc.2c00257. 35776849 PMC9367016

[ref53] Staab J. K.; Chilton N. F. Analytic Linear Vibronic Coupling Method for First-Principles Spin- Dynamics Calculations in Single-Molecule Magnets. J. Chem. Theory Comput. 2022, 18, 6588–6599. 10.1021/acs.jctc.2c00611. 36269220 PMC9648194

[ref54] Egidi F.; Bloino J.; Cappelli C.; Barone V.; Tomasi J. Tuning of NMR and EPR parameters by vibrational averaging and environmental effects: an integrated computational approach. Mol. Phys. 2013, 111, 1345–1354. 10.1080/00268976.2013.796413.

[ref55] Lang L. C.; Ravera E.; Parigi G.; Luchinat C.; Neese F. Theoretical analysis of the long-distance limit of NMR chemical shieldings. J. Chem. Phys. 2022, 156, 154115 10.1063/5.0088162. 35459319

[ref56] Lang L.; Ravera E.; Parigi G.; Luchinat C.; Neese F. Solution of a Puzzle: High-Level Quantum-Chemical Treatment of Pseudocontact Chemical Shifts Confirms Classic Semiempirical Theory. J. Phys. Chem. Lett. 2020, 11, 8735–8744. 10.1021/acs.jpclett.0c02462. 32930598 PMC7584370

[ref57] Glasbrenner M.; Vogler S.; Ochsenfeld C. Linear and sublinear scaling computation of the electronic g-tensor at the density functional theory level. J. Chem. Phys. 2019, 150, 024104 10.1063/1.5066266. 30646705

[ref58] Glasbrenner M.; Vogler S.; Ochsenfeld C. Gauge-origin dependence in electronic g-tensor calculations. J. Chem. Phys. 2018, 148, 214101 10.1063/1.5028454. 29884060

[ref59] Kaupp M.; Remenyi C.; Vaara J.; Malkina O. L.; Malkin V. G. Density Functional Calculations of Electronic g-Tensors for Semiquinone Radical Anions. The Role of Hydrogen Bonding and Substituent Effects. J. Am. Chem. Soc. 2002, 124, 2709–2722. 10.1021/ja0162764. 11890822

[ref60] Kossmann S.; Kirchner B.; Neese F. Performance of modern density functional theory for the prediction of hyperfine structure: meta-GGA and double hybrid functionals. Mol. Phys. 2007, 105, 2049–2071. 10.1080/00268970701604655.

[ref61] Houriez C.; Masella M.; Ferré N. Structural and atoms-in-molecules analysis of hydrogen-bond network around nitroxides in liquid water. J. Chem. Phys. 2010, 133, 124508 10.1063/1.3478999. 20886951

[ref62] Houriez C.; Ferré N.; Siri D.; Masella M. Further Insights into the Environmental Effects on the Computed Hyperfine Coupling Constants of Nitroxides in Aqueous Solution. J. Phys. Chem. B 2009, 113, 15047–15056. 10.1021/jp906828v. 19845322

[ref63] Houriez C.; Ferré N.; Masella M.; Siri D. Prediction of nitroxide hyperfine coupling constants in solution from combined nanosecond scale simulations and quantum computations. J. Chem. Phys. 2008, 128, 244504 10.1063/1.2939121. 18601346

[ref64] Hecker F.; Fries L.; Hiller M.; Chiesa M.; Bennati M. ^17^ O Hyperfine Spectroscopy Reveals Hydration Structure of Nitroxide Radicals in Aqueous Solutions. Angew. Chem., Int. Ed. 2022, 62, e202213700 10.1002/anie.202213700. PMC10107301 36399425

[ref65] Puzzarini C.; Barone V. Toward spectroscopic accuracy for organic free radicals: Molecular structure, vibrational spectrum, and magnetic properties of F2NO. J. Chem. Phys. 2008, 129, 084306 10.1063/1.2969820. 19044822

[ref66] Saitow M.; Neese F. Accurate spin-densities based on the domain-based local pair-natural orbital coupled-cluster theory. J. Chem. Phys. 2018, 149, 034104 10.1063/1.5027114. 30037259

[ref67] Riplinger C.; Pinski P.; Becker U.; Valeev E. F.; Neese F. Sparse maps—A systematic infrastructure for reduced-scaling electronic structure methods. II. Linear scaling domain based pair natural orbital coupled cluster theory. J. Chem. Phys. 2016, 144, 024109 10.1063/1.4939030. 26772556

[ref68] Pinski P.; Riplinger C.; Valeev E. F.; Neese F. Sparse maps-A systematic infrastructure for reduced-scaling electronic structure methods. I. An efficient and simple linear scaling local MP2 method that uses an intermediate basis of pair natural orbitals. J. Chem. Phys. 2015, 143, 034108 10.1063/1.4926879. 26203015

[ref69] Saitow M.; Becker U.; Riplinger C.; Valeev E. F.; Neese F. A new near-linear scaling, efficient and accurate, open-shell domain-based local pair natural orbital coupled cluster singles and doubles theory. J. Chem. Phys. 2017, 146, 164105 10.1063/1.4981521. 28456208

[ref70] Sharma B.; Tran V. A.; Pongratz T.; Galazzo L.; Zhurko I.; Bordignon E.; Kast S. M.; Neese F.; Marx D. A Joint Venture of Ab Initio Molecular Dynamics, Coupled Cluster Electronic Structure Methods, and Liquid-State Theory to Compute Accurate Isotropic Hyperfine Constants of Nitroxide Probes in Water. J. Chem. Theory Comput. 2021, 17, 6366–6386. 10.1021/acs.jctc.1c00582. 34516119 PMC8515807

[ref71] Cimino P.; Neese F.; Barone V. Calculation of Magnetic Tensors and EPR Spectra for Free Radicals in Different Environments. In Computational Spectroscopy, 1st ed.; Grunenberg J., Ed.; Wiley, 2010; pp 63–104.

[ref72] Pavone M.; Cimino P.; De Angelis F.; Barone V. Interplay of Stereoelectronic and Enviromental Effects in Tuning the Structural and Magnetic Properties of a Prototypical Spin Probe: Further Insights from a First Principle Dynamical Approach. J. Am. Chem. Soc. 2006, 128, 4338–4347. 10.1021/ja0574872. 16569010

[ref73] Savitsky A.; Dubinskii A. A.; Plato M.; Grishin Y. A.; Zimmermann H.; Möbius K. High-Field EPR and ESEEM Investigation of the Nitrogen Quadrupole Interaction of Nitroxide Spin Labels in Disordered Solids: Toward Differentiation between Polarity and Proticity Matrix Effects on Protein Function. J. Phys. Chem. B 2008, 112, 9079–9090. 10.1021/jp711640p. 18593147

[ref74] Volodarskii L. B.; Reznikov V.; Kobrin V. Preparation and Properties of Imidazolinium Salts Containing a Nitroxyl Radical Center. J. Org. Chem. 1979, 15, 415–422.

[ref75] Volodarskii L.; Reznikov V.; Kobrin V. Preparation and Properties of Imidazolinium Salts Containing a Nitroxyl Radical Center. Chem. Abstr. 1982, 91, 5158w.

[ref76] Sevast’yanova T. K.; Volodarskii L. B. Preparation of stable iminoxyl radicals of 3-imidazolines. Bull. Russ. Acad. Sci.: Phys. 1972, 21, 2276–2278. 10.1007/bf00855320.

[ref77] Margita K.; Voinov M. A.; Smirnov A. I. Effect of Solution Ionic Strength on the pK(a) of the Nitroxide pH EPR Probe 2,2,3,4,5,5-Hexamethylimidazolidin-1-oxyl. Cell Biochem. Biophys. 2017, 75, 185–193. 10.1007/s12013-017-0780-y. 28210984

[ref78] Stoll S.; Schweiger A. EasySpin, a comprehensive software package for spectral simulation and analysis in EPR. J. Magn. Reson. 2006, 178, 42–55. 10.1016/j.jmr.2005.08.013. 16188474

[ref79] Gast P.; Groenen E. J. J. EPR Interactions - g -Anisotropy. In eMagRes; Harris R. K., Wasylishen R. L., Eds.; John Wiley & Sons, Ltd: Chichester, UK, 2016; pp 1435–1444.

[ref80] Marx D.; Hutter J. Ab Initio Molecular Dynamics: Basic Theory and Advanced Methods; Cambridge University Press, 2009.

[ref81] Adamo C.; Barone V. Toward reliable density functional methods without adjustable parameters: The PBE0 model. J. Chem. Phys. 1999, 110, 6158–6170. 10.1063/1.478522.

[ref82] Zhang Y.; Yang W. Comment on “Generalized Gradient Approximation Made Simple”. Phys. Rev. Lett. 1998, 80, 890 10.1103/physrevlett.80.890.

[ref83] Grimme S.; Antony J.; Ehrlich S.; Krieg H. A consistent and accurate ab initio parametrization of density functional dispersion correction (DFT-D) for the 94 elements H-Pu. J. Chem. Phys. 2010, 132, 154104 10.1063/1.3382344. 20423165

[ref84] Hutter J.; Iannuzzi M.; Schiffmann F.; VandeVondele J. cp2k: atomistic simulations of condensed matter systems. Wiley Interdiscip. Rev.: Comput. Mol. Sci. 2014, 4, 15–25. 10.1002/wcms.1159.

[ref85] Kühne T. D.; Iannuzzi M.; Del Ben M.; Rybkin V. V.; Seewald P.; Stein F.; Laino T.; Khaliullin R. Z.; Schütt O.; Schiffmann F.; et al. CP2K: An electronic structure and molecular dynamics software package - Quickstep: Efficient and accurate electronic structure calculations. J. Chem. Phys. 2020, 152, 194103 10.1063/5.0007045. 33687235

[ref86] Guidon M.; Hutter J.; VandeVondele J. Auxiliary Density Matrix Methods for Hartree–Fock Exchange Calculations. J. Chem. Theory Comput. 2010, 6, 2348–2364. 10.1021/ct1002225. 26613491

[ref87] Pronk S.; Páll S.; Schulz R.; Larsson P.; Bjelkmar P.; Apostolov R.; Shirts M. R.; Smith J. C.; Kasson P. M.; van der Spoel D.; Hess B.; Lindahl E. GROMACS 4.5: a high-throughput and highly parallel open source molecular simulation toolkit. Bioinform 2013, 29, 845–854. 10.1093/bioinformatics/btt055. PMC3605599 23407358

[ref88] Genovese L.; Deutsch T.; Neelov A.; Goedecker S.; Beylkin G. Efficient solution of Poisson’s equation with free boundary conditions. J. Chem. Phys. 2006, 125, 074105 10.1063/1.2335442. 16942320

[ref89] Genovese L.; Deutsch T.; Goedecker S. Efficient and accurate three-dimensional Poisson solver for surface problems. J. Chem. Phys. 2007, 127, 054704 10.1063/1.2754685. 17688354

[ref90] Neese F.; Wennmohs F.; Becker U.; Riplinger C. The ORCA quantum chemistry program package. J. Chem. Phys. 2020, 152, 224108 10.1063/5.0004608. 32534543

[ref91] Huang J.; Rauscher S.; Nawrocki G.; Ran T.; Feig M.; de Groot B. L.; Grubmüller H.; MacKerell A. D. CHARMM36: An improved force field for folded and intrinsically disordered proteins. Biophys. J. 2017, 112, 175a–176a. 10.1016/j.bpj.2016.11.971. PMC5199616 27819658

[ref93] Hölzl C.; Kibies P.; Imoto S.; Frach R.; Suladze S.; Winter R.; Marx D.; Horinek D.; Kast S. M. Design principles for high-pressure force fields: Aqueous TMAO solutions from ambient to kilobar pressures. J. Chem. Phys. 2016, 144, 144104 10.1063/1.4944991. 27083705

[ref94] Imoto S.; Forbert H.; Marx D. Water structure and solvation of osmolytes at high hydrostatic pressure: pure water and TMAO solutions at 10 kbar versus 1 bar. Phys. Chem. Chem. Phys. 2015, 17, 24224–24237. 10.1039/c5cp03069b. 26325021

[ref95] Saitta A. M.; Datchi F. Structure and phase diagram of high-density water: The role of interstitial molecules. Phys. Rev. E: Stat., Nonlinear, Soft Matter Phys. 2003, 67, 020201 10.1103/physreve.67.020201. 12636643

[ref96] Yan Z.; Buldyrev S. V.; Kumar P.; Giovambattista N.; Debenedetti P. G.; Stanley H. E. Structure of the first- and second-neighbor shells of simulated water: Quantitative relation to translational and orientational order. Phys. Rev. E: Stat., Nonlinear, Soft Matter Phys. 2007, 76, 051201 10.1103/physreve.76.051201. 18233643

[ref97] Kumar N.; Marx D. How do ribozymes accommodate additional water molecules upon hydrostatic compression deep into the kilobar pressure regime?. Biophys. Chem. 2019, 252, 106192 10.1016/j.bpc.2019.106192. 31173927

[ref98] Imoto S.; Marx D. How Can Protons Migrate in Extremely Compressed Liquid Water?. Phys. Rev. Lett. 2020, 125, 086001 10.1103/physrevlett.125.086001. 32909792

[ref99] Kirilina E. P.; Prisner T. F.; Bennati M.; Endeward B.; Dzuba S. A.; Fuchs M. R.; Möbius K.; Schnegg A. Molecular dynamics of nitroxides in glasses as studied by multi-frequency EPR. Magn. Reson. Chem. 2005, 43, S119–S129. 10.1002/mrc.1677. 16235207

[ref100] Pavone M.; Cimino P.; Crescenzi O.; Sillanpää A.; Barone V. Interplay of Intrinsic, Environmental, and Dynamic Effects in Tuning the EPR Parameters of Nitroxides: Further Insights from an Integrated Computational Approach. J. Phys. Chem. B 2007, 111, 8928–8939. 10.1021/jp0727805. 17608525

[ref101] More C.; Bertrand P.; Gayda J. P. Simulation of the EPR spectra of metalloproteins based on a physical description of the “g-strain the vendors ha ” effect. J. Magn. Reson. 1987, 73, 13–22. 10.1016/0022-2364(87)90221-6.

