# Supplementary material for: Dissecting the Molecular Origin of g-Tensor Heterogeneity and Strain in Nitroxide Radicals in Water: Electron Paramagnetic Resonance Experiment versus Theory
Source: J Phys Chem A. 2023 Jul 31;127(31):6447–66. doi: 10.1021/acs.jpca.3c02879 (PMC10424240; doi:10.1021/acs.jpca.3c02879)
Supplement: Supplementary file 1 — jp3c02879_si_001.pdf [file jp3c02879_si_001.pdf]

Dissecting the Molecular Origin of g-Tensor Hetero-geneity and Strain in Nitroxide Radicals in Water: Electron Paramagnetic Resonance Experiment vs. Theory

Van Anh Tran[‡a], Markus Teucher[‡b], Laura Galazzo[‡cd], Bikramjit Sharma[‡e], Tim Pongratz[f], Stefan M. Kast[f], Dominik Marx[e]*, Enrica Bordignon[cd]*, Alexander Schnegg[b]*, Frank Neese[a]**

*[‡] contributed equally, * corresponding authors*

a: Max-Planck-Institut für Kohlenforschung, Kaiser-Wilhelm-Platz 1, 45470 Mülheim an der Ruhr, Germany

b: Max-Planck-Institut für Chemische Energiekonversion, Stiftstraße 34-36, 45470 Mülheim an der Ruhr, Germany

c: Department of Physical Chemistry, University of Geneva, Quai Ernest Ansermet 30, 1211 Geneva, Switzerland

d: Faculty of Chemistry and Biochemistry, Ruhr-Universität Bochum, 44780 Bochum, Germany

e: Lehrstuhl für Theoretische Chemie, Ruhr-Universität Bochum, 44780 Bochum, Germany

f: Fakultät für Chemie und Chemische Biologie, Technische Universität Dortmund, Otto-Hahn-Str. 4a, 44227 Dortmund, Germany

Supplementary Information

1. Example of W-band spectra analysis

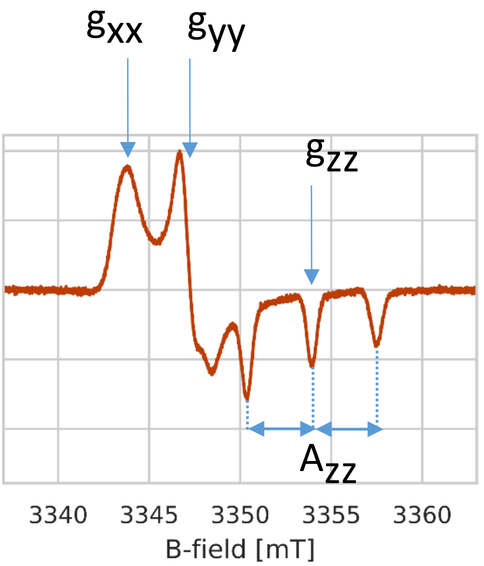

Figure S1. Low temperature cw W-band EPR spectrum of a nitroxide, with the characteristic g_{xx}, g_{yy}, g_{zz} and A_{zz} *parameters indicated*.

2. Experimental spectra

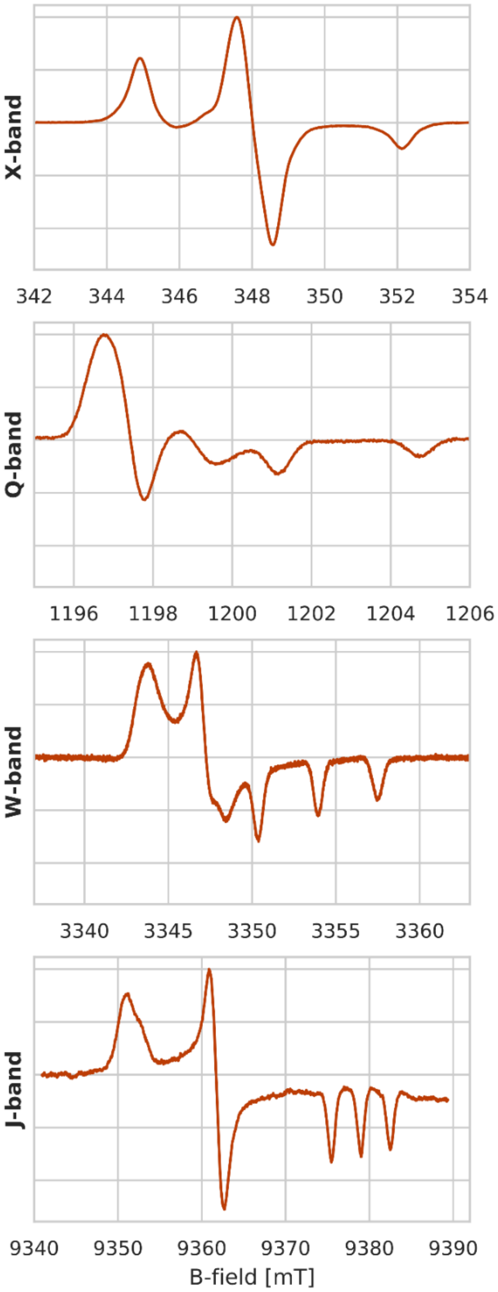

Figure S2. Multifrequency low temperature cw spectra of HMI in water at pH 10. The B-field axis was calibrated following the procedure described. Measurement parameters are given in the *EPR experimental parameters* section of the manuscript. The poor resolution of the gxx populations in the W-band spectrum is possibly due to inter- and intramolecular proton-hyperfine interactions, which are the most probable source of the field-independent contribution to the inhomogeneous linewidth in frozen solution nitroxide EPR spectra. To investigate the influence of intermolecular proton-hyperfine interactions due to the solvent, W-band EPR measurements were also performed in deuterated water (not shown). However, the reduction in hyperfine interaction with solvent molecules has no discernible effect on the linewidth.

3. Magnetic Field Calibration

To achieve a consistent magnetic field calibration across the different frequency bands used in this work, the unique properties of two standard samples were exploited. To calibrate the linearity of the magnetic field, a Mn^{2+} in ZnS sample (WLG.Artis, Germany) was used, taking advantage of its 6 distinct lines distributed over a wide magnetic field range (≈ 34 mT). Complementary, a carbon fiber standard sample[1] with a single sharp line was used to calibrate the absolute magnetic field position after linearization. The EPR parameters of both samples are listed in Table S0.

Table S0. EPR parameters of the standard samples.

sample	g	A [MHz]
Mn^{2+} in ZnS	2.0024 ± 0.0003	190.68
carbon fiber[1]	2.002644 ± 0.000015	n.a.

Alongside each acquired HMI spectrum, the spectra of both standard samples were measured using identical acquisition parameters. Specifically, this entails the sweep range, number of points and time constant to ensure a constant sweep rate across the magnetic field range of interest. At X- and Q-band no measurable hysteresis effects of the utilized electromagnet on the spectra could be found, while at W- and J-band the superconducting magnets exhibited strong hysteresis effects. To circumvent the influence of these effects on the spectra, the magnets were always brought to the highest magnetic field reachable for the sweep coils before starting the acquisition of a spectrum. Spectra were then recorded by sweeping from low to high magnetic field values.

The magnetic field calibration of the HMI cw EPR spectra was subsequently performed using the following routine:

1. Mn^{2+} in ZnS sample

1.1 Simulate Mn^{2+} in ZnS spectrum based on the utilized experimental parameters with the MATLAB routine “pepper” from EasySpin (version 5.2.33)[2].

1.2 Determine the peak positions of both the simulated and experimentally recorded Mn^{2+} in ZnS spectra.

1.3 Create a calibration curve by fitting the difference between the peak positions ΔB determined in 1.2 versus the magnetic field axis of the simulation using a suitable model.
This corresponds to the deviation from the linearity of the magnetic field sweep.

1.4 Correct the experimental Mn^{2+} in ZnS spectrum.

2. Carbon Fiber sample

2.1 Correct the experimental spectrum of the carbon fiber sample with the in 1.3 determined calibration curve.

2.2 Simulate carbon fiber spectrum based on the utilized experimental parameters with the MATLAB routine “pepper” from EasySpin (version 5.2.33)[2].

2.3 Determine the difference in the peak position of both the simulated and experimentally recorded carbon fiber spectra.

This corresponds to the error in the determination of the absolute magnetic field position.

3. HMI sample

3.1 Correct the experimental HMI spectrum with the in 1.3 determined calibration curve.

3.2 Shift the HMI spectrum by the in 2.3 determined offset.

The spectrum has now a calibrated magnetic field axis, both with respect to linearity, as well as absolute field position.

4. Explanation of H-bond criterion

In Figure 4 of the main text, the upper left inset schematically shows two geometrical parameters $r_{O...H}$ and θ used to define HMI-water H-bond. In the upper right inset, there is a contour with $r_{O...H}$ in x-axis and cos(θ) on y-axis. The red line on the contour is $r_{O...H}$ = -1.71cosθ+1.37, which defines the separatrix according to our H-bond criterion. This means the red line sets the upper limit of the H-bond criterion. The region below this red line corresponds to hydrogen bonding, i.e. all the $r_{O...H}$ and cos(θ) corresponding to the probability region in the contour plot below the red line satisfy the H-bond criterion. Thus, for a given snapshot, if one (or n number of) water molecule(s) has (have) $r_{O...H}$ and θ values such that $r_{O...H}$ < -1.71cosθ+1.37, then automatically, it corresponds to some point in the bottom-left region with respect to the red separatrix line which implies H-bonding. Below, we compare the H-bond populations obtained from our criterion to those based the widely used so-called "Luzar-Chandler criterion"[3,4] and its improved version (the "Relaxed Luzar-Chandler criterion")[4].

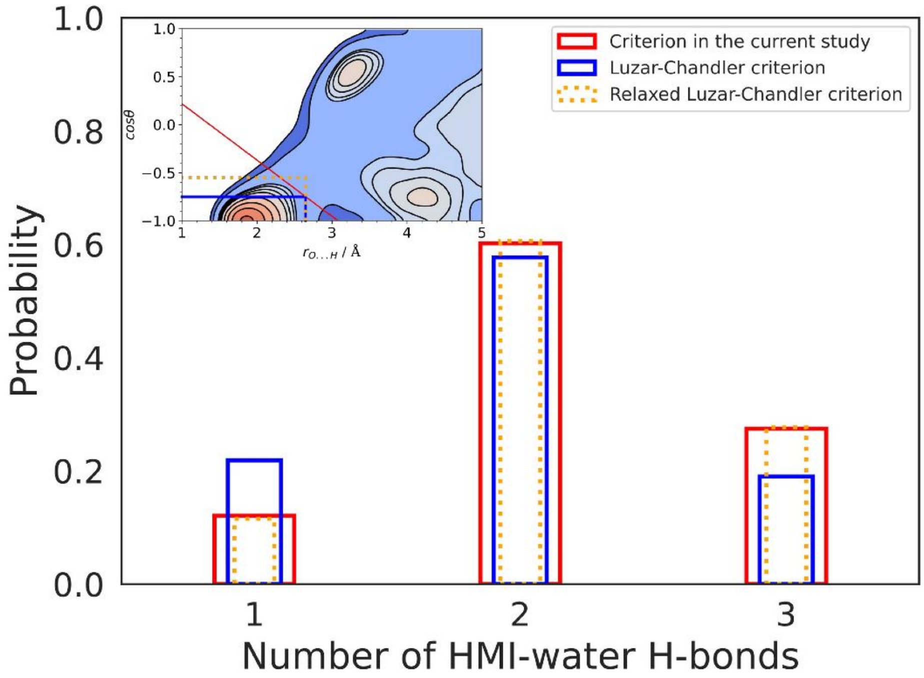

Figure S3: Comparison of the hydrogen bond criterion used in this study to the stricter Luzar-Chandler criterion ($r_{O\cdots H} < 2.65$ Å and $\theta_{O\cdots O-H} < 30°$) and its relaxed version ($r_{O\cdots H} < 2.65$ Å and $\theta_{O\cdots O-H} < 40°$), respectively, see text for references. The cutoff for $r_{O\cdots H}$ is obtained from the first minimum of the radial distribution functions of the

HMI oxygen and the water hydrogens. The inset shows these criteria projected on the corresponding joint probability distribution function where the rectangular areas marked by solid blue and dotted orange lines denote those configurations considered to be hydrogen bonded according to the Luzar-Chandler and Relaxed Luzar-Chandler criteria. Our refined definition fully encompasses the entire hydrogen bonding region while the original Luzar-Chandler criterion captures only a part of it. Improving it, led to the Relaxed Luzar-Chandler criterion, which now captures a major chunk of the missing hydrogen-bonding region in the former. The refined criterion provides hydrogen bond populations, see main graph, that are very close to the ones obtained from our criterion (whereas the original Luzar-Chandler criterion deviates from both). Still, the average numbers of interstitial water molecules are very close to each other (namely 0.70, 0.87 and 0.69 according to our, Luzar-Chander and Relaxed Luzar-Chandler criteria, respectively), implying that the splitting into H-bonded and interstitial water molecules around the HMI oxygen site is rather independent of the specific criterion used.

5. Dihedral angle distribution

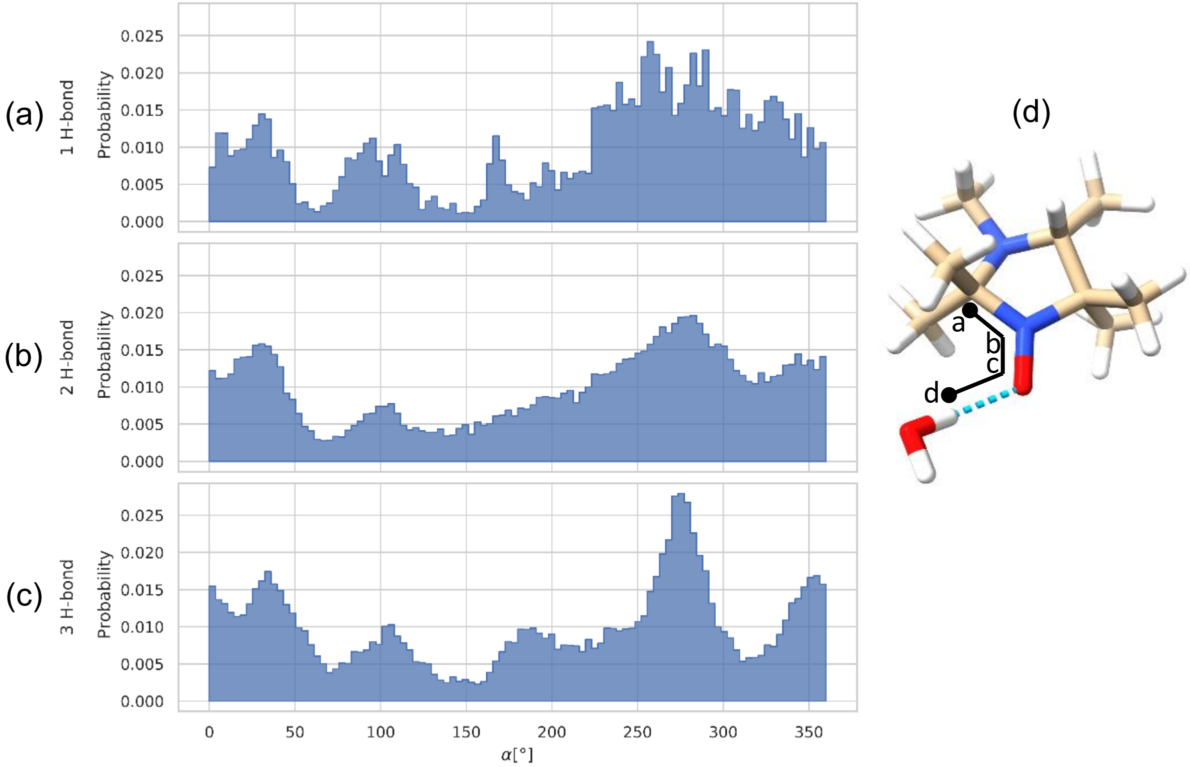

Figure S4. Distribution of dihedral angle α for (a) 1, (b) 2 and (c) 3 H-bond subensembles. The dihedral angle α is defined by the planes containing atoms CNO and NOH where H is the hydrogen atom of the hydrogen bonded water. A schematic of the planes (abc and bcd containing CNO and NOH atoms, respectively) is shown in (d).

6. Dihedral angle versus g_{xx}

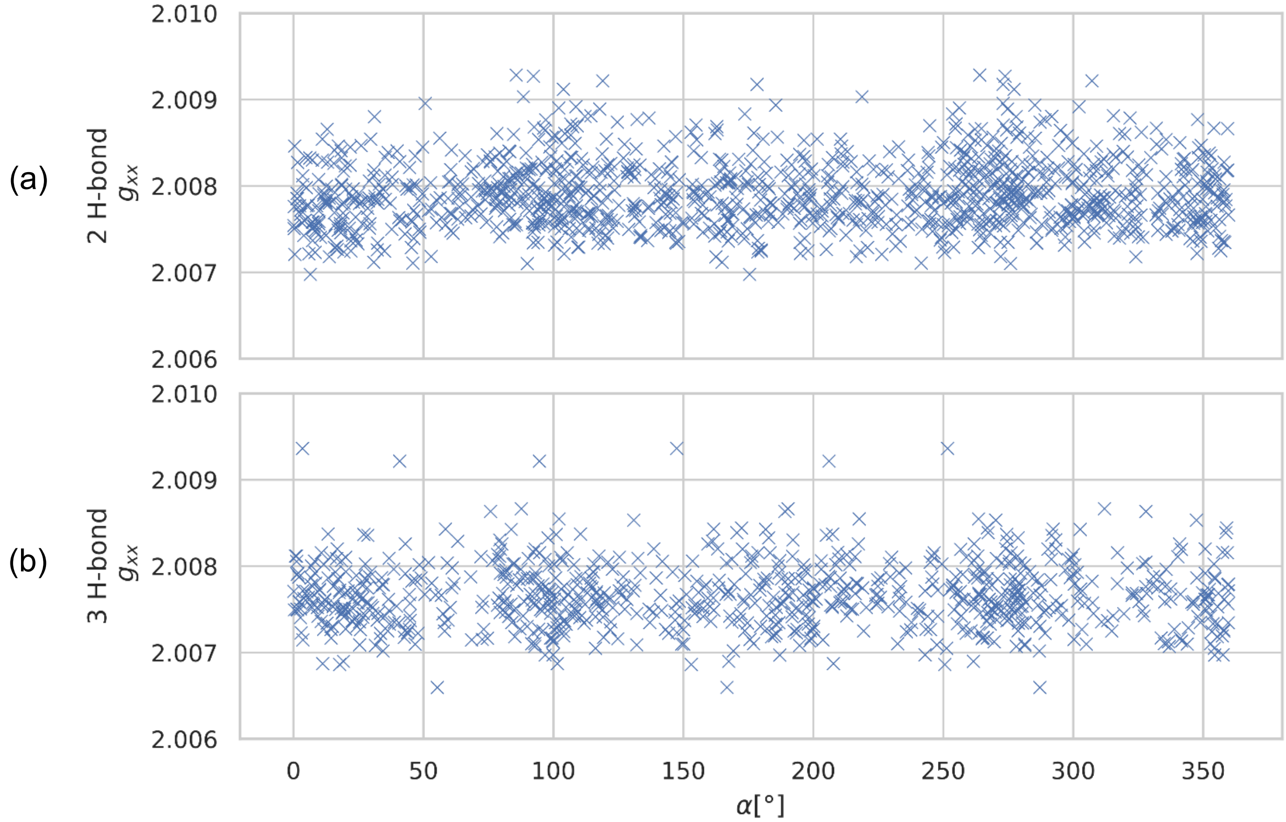

Figure S5. Variation of g_{xx} with respect to dihedral angle α (as shown in Figure S3(d)) for (a) 2 and (b) 3 H-bond subensembles. The g_{xx} values are scattered over the entire possible range for a given value of α, meaning that the two quantities have no correlation.

7. Solvation Modeling: AIMD vs. EC-RISM

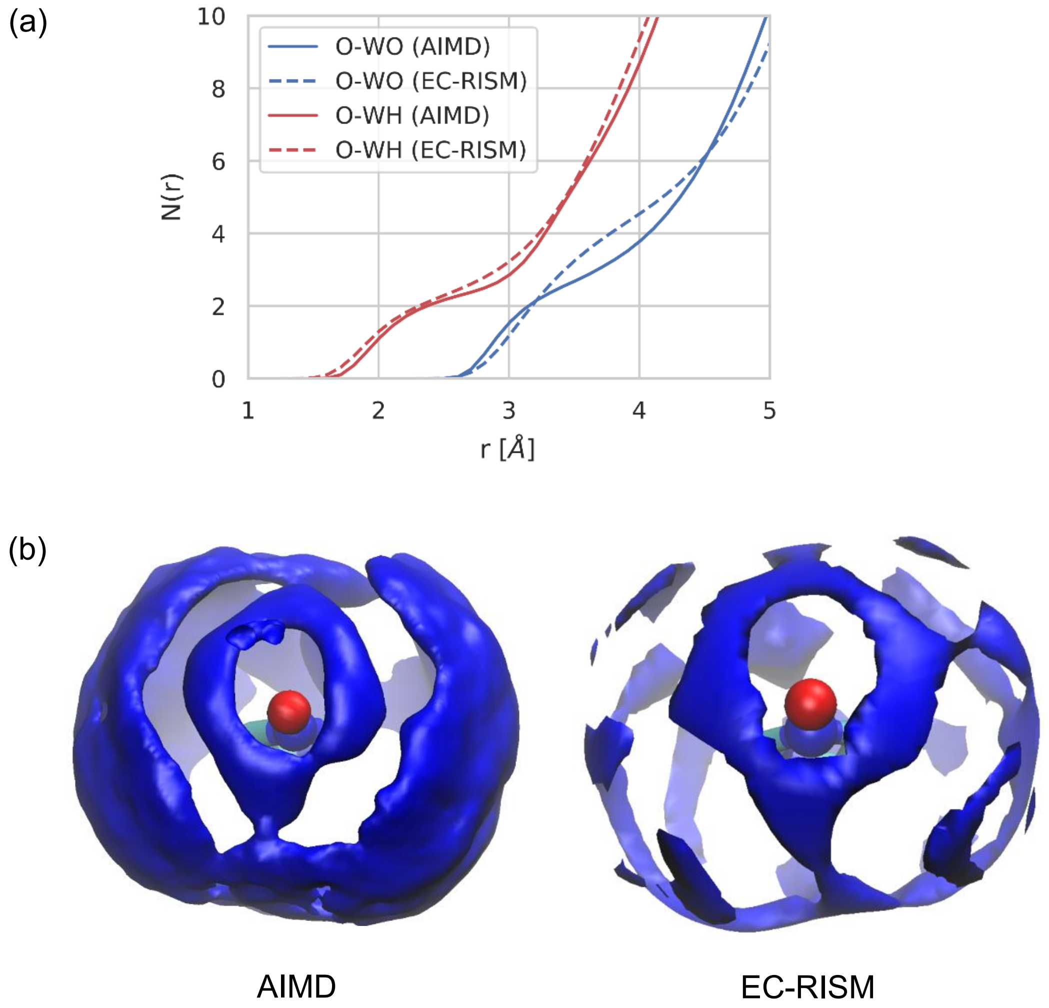

Figure S6. (a) Comparison of running coordination number of water oxygen/hydrogen (WO/WH) with respect to HMI oxygen (O) as obtained from AIMD and EC-RISM. (b) Qualitative comparison of solvation of HMI oxygen as modeled by AIMD and EC-RISM in terms of spatial distribution functions of WO (in red color). Both methods give spatially symmetric water density around HMI oxygen.

8. Theoretical simulated EC-RISM spectra versus experiment

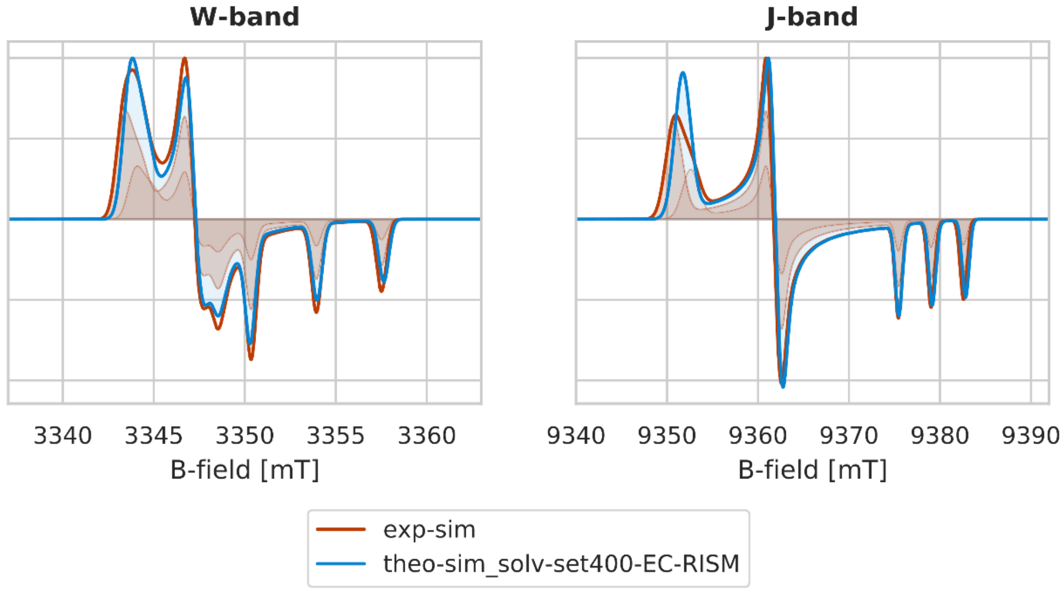

Figure S7. Analogue of Figure 8 in main text with **theo-sim** spectra of the solv-set400-EC-RISM. Since there is no differentiation between specific H-bond situations in the EC-RISM treatment of solvation, there is only one spectral component in contrast to the QM/MM approach.

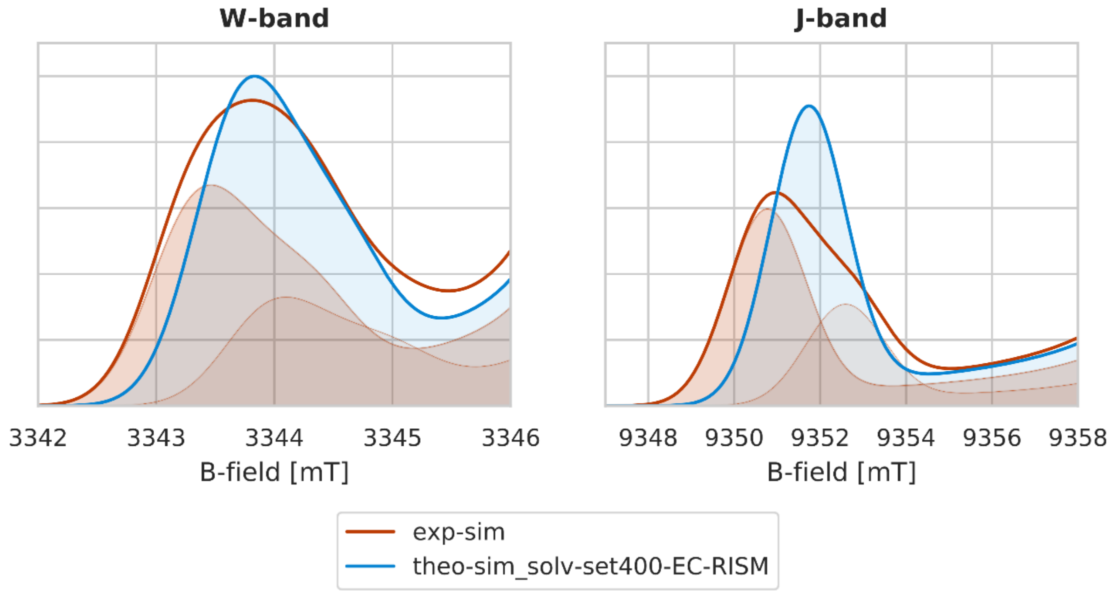

Figure S8. Analogue of Figure 9 in main text with **theo-sim** spectra of solv-set400-EC-RISM.

9. Theoretical spectra at different level of theory

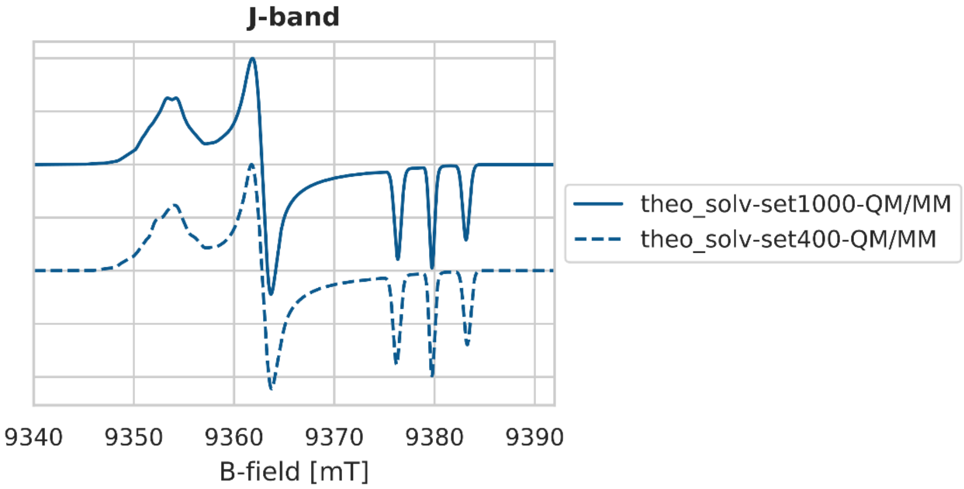

Figure S9. Comparison of theoretical spectra for different solvated data sets.

10. EC-RISM: theo vs. theo-sim

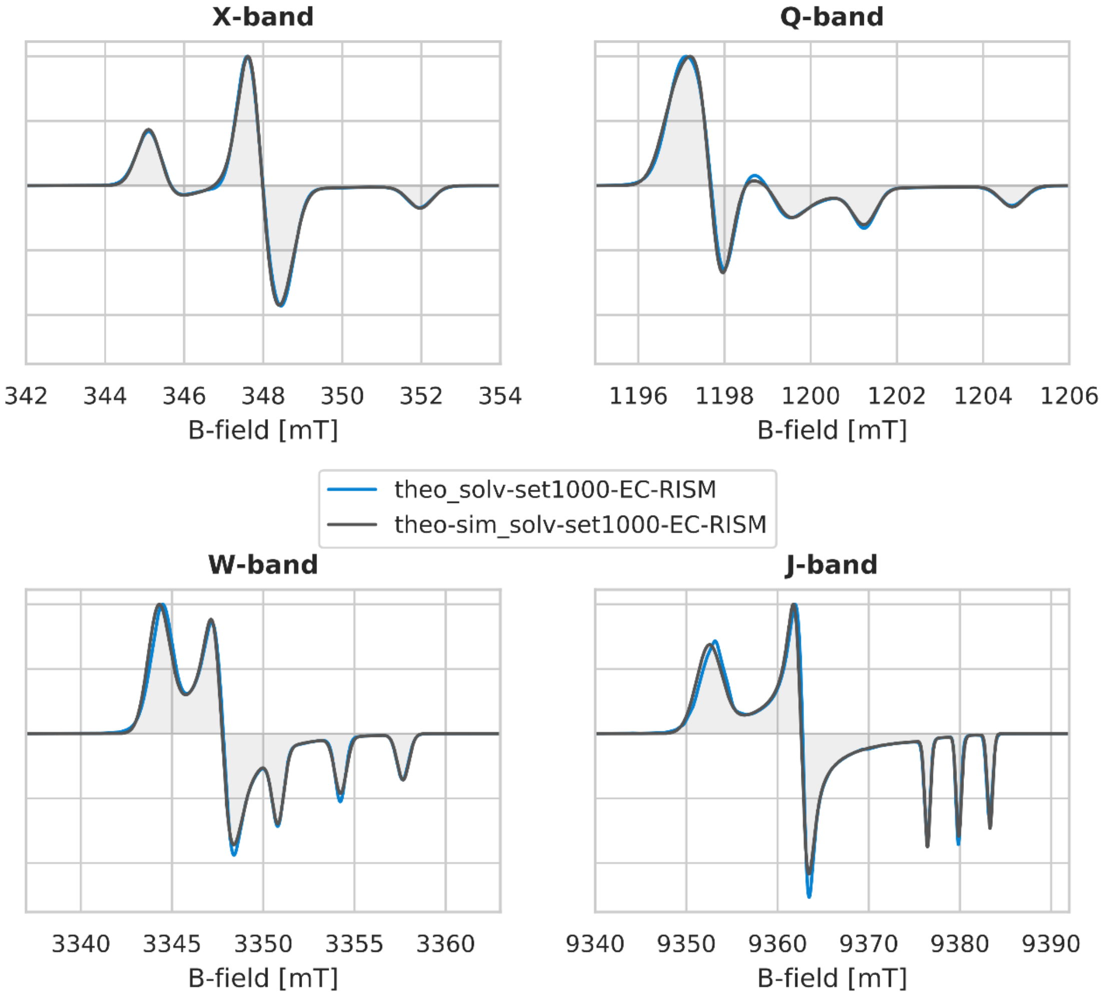

Figure S10. Analogue of Figure 11 in main text with solv-set-1000-EC-RISM. Since there is no differentiation between specific H-bond situations in the EC-RISM treatment of solvation, there is only one spectral component in contrast to the QM/MM approach.

11. Theoretical linewidth analysis

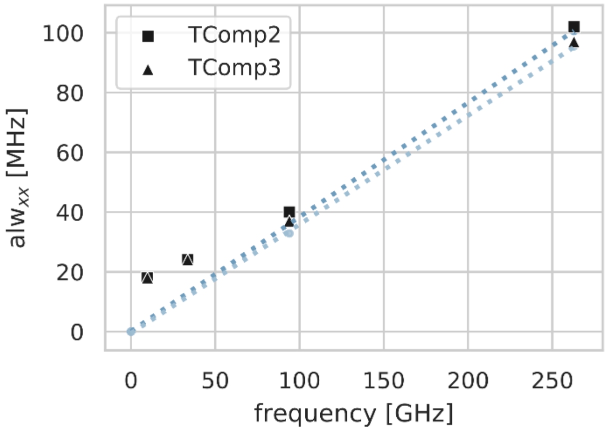

Figure S11. Plot of the apparent linewidth alw$_{xx}$ as obtained by the fits of the multifrequency spectra (**theo**) vs. measuring frequency for the ***TComp2*** and ***TComp3*** components, corresponding to the 2 and 3 H-bond subsets of solv-set1000-QM/MM.

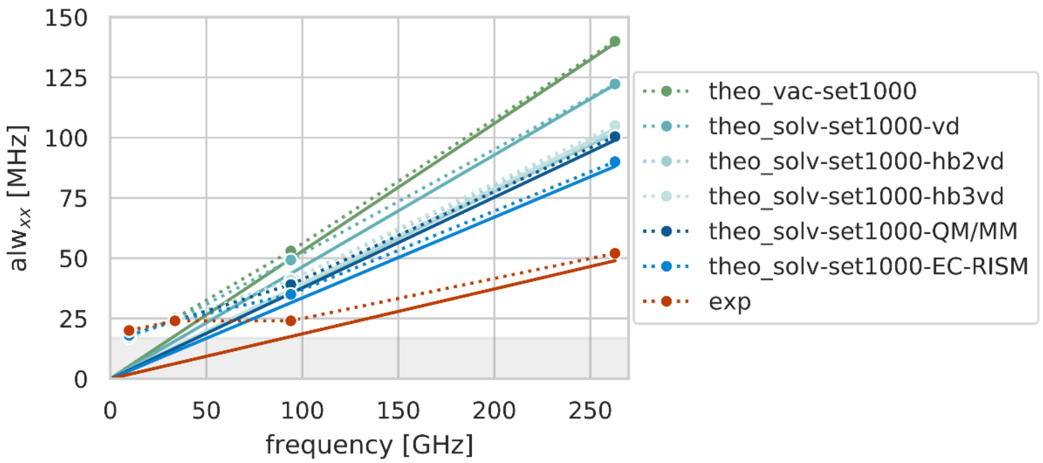

Figure S12. Apparent linewidth alw$_{xx}$ vs. spectrometer frequency for all different data sets based on the solvated (solv-set1000) or vacuum (vac-set1000) trajectory vs. experimental data. The environment of the solvated data set was either included explicitly based on the QM/MM scheme or removed by vertically desolvating the snapshots. The addition "hb2vd" and "hb3vd" refer to the subsets of solv-set1000 that consist of the partially vertically desolvated clusters of HMI by keeping all water molecules forming 2 and 3 H-bonds towards HMI, respectively.

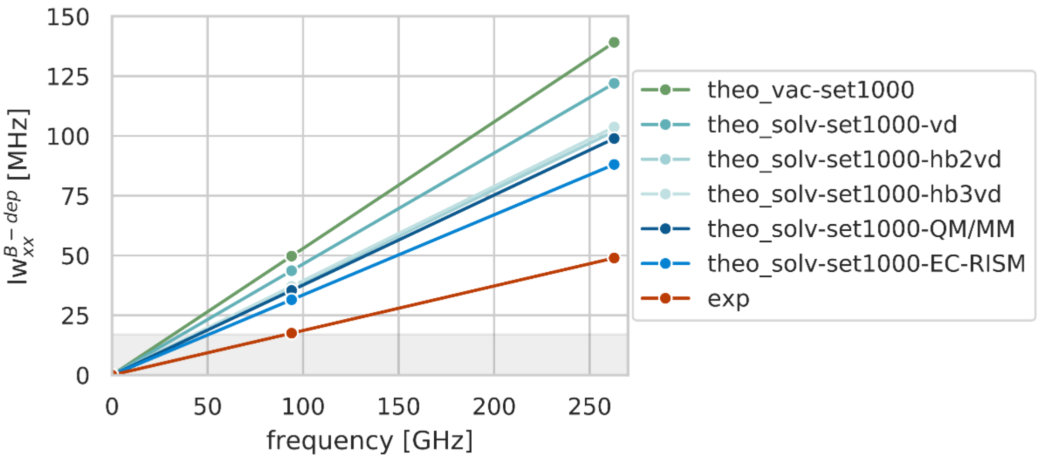

Figure S13. Linear regression of field-dependent linewidth contribution to fitted alw_{xx}. Data points that were used for fitting and obtained slope are given in Table S1.

Table S1. Data points used for linear regression of field-dependent lw_{xx}^{B-dep} of different theoretical spectra in comparison with experiment. lw_{xx}^{B-dep} was computed according to equation 1 given in the main text assuming a field-independent linewidth of 17 MHz as estimated from experiment. The slopes of the regression fits are given in ppm.

	exp	Theo_solv-set1000-EC-RISM	Theo_solv-set1000-QM/MM	Theo_solv-set1000-hb2vd	Theo_solv-set1000-hb3vd	Theo_solv-set1000-vd	Theo_vac-set1000
lw_{xx}^{B-dep} at 0 GHz	0	0	0	0	0	0	0
lw_{xx}^{B-dep} at W-band	17	31	35	37	37	46	50
lw_{xx}^{B-dep} at J-band	49	88	99	102	104	121	139
slope	186	335	376	388	395	464	529
R^2	0.9999	0.9999	1.0000	0.9999	1.0000	0.9995	1.0000

The consideration of additional distinct vertically desolvated ensembles, namely theo-solv-set1000-hb2vd and theo-solv-set1000-hb3vd, gives further insights into the theoretical linewidth analysis. The comparison of TComp2 (table 4 in the main text) vs theo-solv-set1000-hb2vd or TComp3 (table 4 in the main text) vs theo-solv-set1000-hb3vd ensembles where the molecular structure of HMI is same for a given snapshot, shows practically same apparent linewidths (alws), thus the same g-strain, which tells that only the H-bonded water molecules govern the alws of the EPR spectra and other water molecules have no role to play. Furthermore, barely 2 (or 3) H-bonded water molecules are sufficient to drive the alws as no change in alw is observed even in case of fully solvated case (theo-solv-set1000-QM/MM). Thus,

hydrogen bonding alone affects the alws by perturbing the electronic structure of the NO group in solvated state while the remaining water molecules treated within the QM/MM scheme show no effect.

On the other hand, the theo-solv-set1000-vd ensemble has the effects of solvation on the molecular structure of HMI but no perturbation of electronic structure due to formation of H-bonds. Thus, consideration of this ensemble deciphers how the alw, thus the g-strain, is affected by only the molecular structure of HMI as a response to solvation. At higher frequencies (W- and J-band), this ensemble has greater alws than all the solvated ensembles meaning that electronic structure perturbation by H-bonding contributes to the decrease of the alw, thus the g-strain. However, the theo-solv-set1000-vd shows smaller alws compared to that of the theo-solv-set1000-vacuum ensembles where the former is a structural ensemble of HMI with the solvation memory imprinted in it while the latter is a structural ensemble without any reference to solvation. Thus, solvation in general also decreases the alws, thus the g-strain, indirectly by affecting the structure of HMI. For the theo_solv-set1000-ECRISM calculations, where the vertical desolvated structures were used and solvated by EC-RISM, a similar g-strain, as for the QM/MM scheme, can be observed. This finding further indicates that the addition of solvation is the main driver to decrease the alws compared to the vacuum calculations.

12. Analysis of structural properties of HMI in gas versus liquid phase

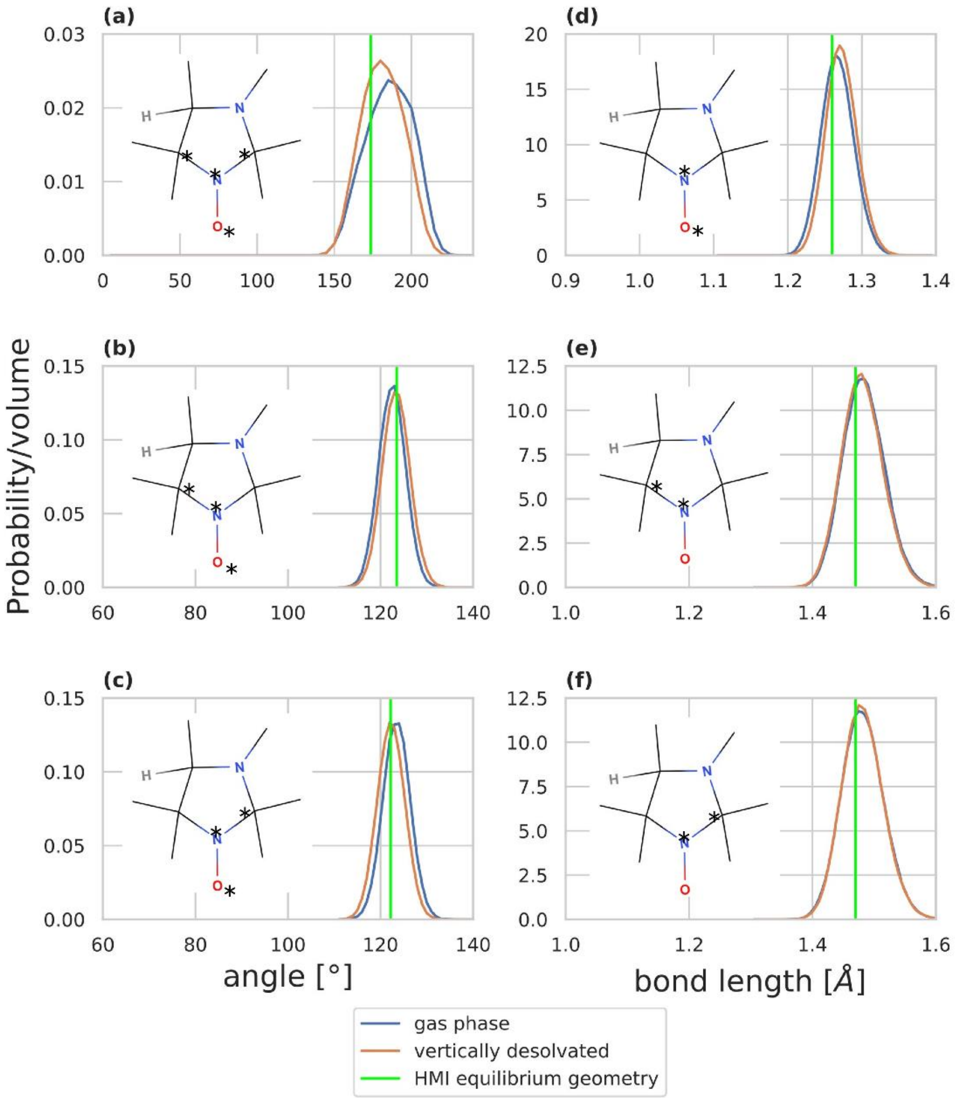

Figure S14: Probability distribution functions of selected structural properties of the HMI molecule around the nitroxy group in the gas phase and in the vertically desolvated state (as sampled from the liquid phase AIMD simulations) both at 300 K. The atoms involved in the angles (left panels) and distances (right panels) are marked with *. (a) Dihedral angle formed by CCNO atoms, (b)/(c) bond angles, (d)/(e)/(f) bond lengths. The data show that the quasi-rigid HMI molecule is structurally not much affected by the liquid-state surrounding compared to the gas phase at the same temperature. This implies that the major contribution to the g-strain is already found in the gas phase as a result of thermal fluctuations that lead to configurational flexibility. The corresponding values of the equilibrium structure of an isolated HMI molecule (shown as vertical green bars) are obtained by geometry optimization of HMI in vacuum using the identical setup as used to carry out the AIMD simulations which provides the consistent static reference structure. The data show that thermal fluctuations (in either gas or liquid phase) not only lead to configurational flexibility as such, but also affect the average structure of HMI.

13.Summary of all theoretical data

The g- and A-values were computed whereas the linewidths were obtained by simulating a fit to the theoretical spectra (**theo**= normalized sum of spectra).

Table S2. Parameters of all theoretically computed and extracted data sets including their subsets (mean values are given).

		QM/MM								EC-RISM		Vertically desolvated			Vertically desolvated H-bond clusters		vacuum
		g- and A-values at revPBE0 level				g-values at revPBE0 level, A-values at DLPNO-CCSD				g- and A-values at revPBE0 level	g-values at revPBE0 level, A-values at DLPNO-CCSD	g- and A-values at revPBE0 level			g- and A-values at revPBE0 level		
subsets (“TComp”)			1 H-bond	2 H-bonds	3 H-bonds		1 H-bond	2 H-bonds	3 H-bonds				2 H-bonds	3 H-bonds	2 H-bonds	3 H-bonds	
No. of snapshots		1000	121	595	270	400	49	249	99	1000	400	1000	595	270	595	270	1000
g_{xx}		2.00788	2.00820	2.00791	2.00767	2.00788	2.00822	2.00788	2.00769	2.00796	2.00794	2.00891	2.00891	2.00893	2.00814	2.00789	2.00886
g_{yy}		2.00576	2.00584	2.00578	2.00572	2.00576	2.00585	2.00575	2.00571	2.00576	2.00577	2.00607	2.00606	2.00608	2.00585	2.00580	2.00604
g_{zz}		2.00214	2.00214			2.00214	2.00214			2.00210	2.00212	2.00215	2.00215	2.00214	2.00214	2.00214	2.00214
A_{xx} [MHz]		7.2	6.5	7.3	7.4	11.2	9.6	11.6	11.0	7.4	12.3	5.2	5.3	4.9	6.9	6.9	6.0
A_{yy} [MHz]		7.5	6.7	7.5	7.7	11.6	9.9	12.0	11.5	7.6	12.8	5.5	5.7	5.3	7.1	7.1	6.3
A_{zz} [MHz]		95.5	91.3	95.2	98.1	98.7	92.6	99.0	101.0	96.1	102.8	82.8	82.8	82.7	92.1	95.0	83.2
alw_{xx} [MHz]	X		18							18			17		17		17
	Q		24							24			24		24		26
	W		43	40	37					35			48	52	41		53
	J		105	102	97					90			121	125	103	105	140
alw_{yy} [MHz]	X		16							17			17		17		15
	Q		21							20			20		21		20
	W		23							24			23		24		25
	J		42	45	47					40			41	42	45	47	42
alw_{zz} [MHz]	X		21							20			20		21		19
	Q		18							18			18		20		19
	W		19							21			18		20		19
	J		20							19			20		20		19

References

1. Konstantin, H.; Tschaggelar, R.; Denninger, G.; Jeschke, G. Double resonance calibration of g factor standards: Carbon fibers as a high precision standard. *J. Magn. Reson.* **2018**, *289*, 100-106.

2. Stoll, S.; Arthur Schweiger, A. EasySpin, a comprehensive software package for spectral simulation and analysis in EPR. *J. Magn. Reson.* **2006**, *178(*1*)*, 42-55.

3. Luzar, A.; Chandler. D. Hydrogen-bond Kinetics in Liquid Water. *Nature* **1996**, *379*, 55-57.

4. Luzar, A. Resolving the Hydrogen Bond Dynamics Conundrum. *J. Chem. Phys.* **2000**, *113*, 10663-10675.
